# Gene expression based survival prediction for cancer patients—A topic modeling approach

**DOI:** 10.1371/journal.pone.0224446

**Published:** 2019-11-15

**Authors:** Luke Kumar, Russell Greiner

**Affiliations:** 1 Department of Computing Science, University of Alberta, Edmonton, Alberta, Canada; 2 Alberta Machine Intelligence Institute (Amii), Edmonton, Alberta, Canada; Politechnika Krakowska im Tadeusza Kosciuszki, POLAND

## Abstract

Cancer is one of the leading cause of death, worldwide. Many believe that genomic data will enable us to better predict the survival time of these patients, which will lead to better, more personalized treatment options and patient care. As standard survival prediction models have a hard time coping with the high-dimensionality of such gene expression data, many projects use some dimensionality reduction techniques to overcome this hurdle. We introduce a novel methodology, inspired by topic modeling from the natural language domain, to derive expressive features from the high-dimensional gene expression data. There, a document is represented as a mixture over a relatively small number of topics, where each topic corresponds to a distribution over the words; here, to accommodate the heterogeneity of a patient’s cancer, we represent each patient (≈ document) as a mixture over cancer-topics, where each cancer-topic is a mixture over gene expression values (≈ words). This required some extensions to the standard LDA model—*e.g*., to accommodate the *real-valued* expression values—leading to our novel *discretized Latent Dirichlet Allocation* (dLDA) procedure. After using this dLDA to learn these cancer-topics, we can then express each patient as a distribution over a small number of cancer-topics, then use this low-dimensional “distribution vector” as input to a learning algorithm—here, we ran the recent survival prediction algorithm, MTLR, on this representation of the cancer dataset. We initially focus on the METABRIC dataset, which describes each of n = 1,981 breast cancer patients using the r = 49,576 gene expression values, from microarrays. Our results show that our approach (dLDA followed by MTLR) provides survival estimates that are more accurate than standard models, in terms of the standard Concordance measure. We then validate this “dLDA+MTLR” approach by running it on the n = 883 Pan-kidney (KIPAN) dataset, over r = 15,529 gene expression values—here using the mRNAseq modality—and find that it again achieves excellent results. In both cases, we also show that the resulting model is calibrated, using the recent “D-calibrated” measure. These successes, in two different cancer types and expression modalities, demonstrates the generality, and the effectiveness, of this approach. The dLDA+MTLR source code is available at https://github.com/nitsanluke/GE-LDA-Survival.

## 1 Introduction

The World Health Organization reports that cancer has become the second leading cause of death globally, as approximately 1 in 6 deaths are caused by some form of cancer [1]. Moreover, cancers are very heterogeneous, in that the outcomes can vary widely for patients with similar diagnoses, who receive the same treatment regimen. This has motivated researchers to seek other features to help predict individual outcomes. Many such analyses use just clinical features. Unfortunately, features such as lymph node status and histological grade, while predictive of metastases, do not appear to be sufficient to reliably categorize clinical outcome [2]. This has led to many efforts to improve the prognosis for cancer, based on genomics data (*e.g*., gene expression (GE) or copy number variation (CNV)), possibly along with the clinical data [2–6]. Focusing for now on breast cancer, van’t Veer *et al*. [2] used the expression of 70 genes to distinguish high vs low risk of distant metastases within five years. Parker *et al*. [4] identified five subtypes of breast cancer, based on a panel of 50 genes (PAM50): luminal A, luminal B, HER2-enriched, basal-like, and normal-like. Later, Curtis *et al*. [7] examined ≈2000 patients from a wide study combining clinical and genomic data, and identified around ten subtypes. All three of these studies showed that their respective subtypes produce significantly different Kaplan-Meier survival curves [8], suggesting such molecular variation does influence the disease progression. There are also many other systems that use such expression information to divide the patients into two categories: high- vs low-risk; *cf*., [2, 6].

More recently, many survival *prediction* models have been applied to cancer cohorts, with the goal of estimating survival times for individual patients; some are based on standard statistical survival analysis techniques, and others based on classic regression algorithms—*e.g*., random survival forests [9] or support vector regression for censored data (SVRc) [10]. With the growing number of gene expression experiments being cataloged for analysis, we need to develop survival prediction models that can utilize such high dimensional data. Our work describes such a system that can learn effective survival prediction models from high-dimensional gene expression data.

The 2012 DREAM Breast Cancer Challenge (BCC) was designed to focus the community’s efforts to improve breast cancer survival prediction [3]. Its organizers made available clinical and genomic data (GE and CNV) of ≈2000 patients from the [7] study (mentioned above). Each submission to the BCC challenge mapped each patient to a single real value (called “risk”), which is predicting that patients with higher risk should die earlier than those with lower risk. The entries were therefore evaluated based on the concordance measure: basically, the percentage of these pairwise predictions that were correct [11]. This is standard, in that many survival prediction tasks use the concordance as the primary measure to assess the performance of the survival predictors, here and in other challenges [12]. The winning model [13] performed statistically better than the state-of-the-art benchmark models [3].

This paper explores several dimensionality reduction technique, including a novel approach based on topic modeling, “discretized Latent Dirichlet Allocation” (dLDA), seeking one that can produce highly predictive features from the high-dimensional gene expression data. We explored several ways to apply this topic-modeling approach to gene expression data, to identify the best ways to use it to map the gene expression description into a much lower dimensional description (from ≈50K features to 30 in this METABRIC dataset). We then gave the resulting transformed data as input to a recently-developed non-parametric learning algorithm, multi-task logistic regression (MTLR), which produced a model that can then predict an individual’s survival distribution [14]. We show that this predictor performs better than other standard survival analysis tools in terms of concordance. We also found that it was “D-calibrated” [15, 16]; see Appendix B.2.

To test the generality of our learning approach (dLDA + MTLR), we then applied the same learning algorithm—the one that worked for the METABRIC microarray gene expression dataset—to the Pan-Kidney dataset, which is a different type of cancer (kidney, not breast), and is described using a different type of features (mRNAseq, not microarray). We found that the resulting predictor was also extremely effective, in terms of both concordance and D-calibration.

This paper provides the following three contributions: (1) We produce an extension to LDA, called “dLDA”, needed to handle continuous data; (2) we use this as input to a survival prediction tool, MTLR—introducing that tool to this bioinformatics community; and (3) we demonstrate that this dLDA+MTLR combination works robustly, in two different datasets, using two different modalities—working better than some other standard approaches, in survival prediction.

Section 2 introduces the basic concepts, related to the survival prediction task in general and latent dirichlet allocation; Section 3 then describes the datasets used in this study; and Section 4 presents an overview of learning and performance tasks, at a high level. Section 5 (resp., 6, 7) then presents our results (resp., discussions, contributions). The supplementary appendices provide additional figures, tables, and other and material—*e.g*., defining some of the terms, and introducing “D-calibration”.

## 2 Foundations

This section provides the foundations: Section 2.1 overviews the survival prediction task in general then Section 2.2 describes Latent Dirichlet Allocation (LDA), first showing its original natural language context, then discussing how we need to extend it for our gene expression context. These significant modifications lead to a discretized variant, dLDA. We also contrast this approach with other survival analysis of gene expressions.

### 2.1 Survival prediction

**Fig 1:**
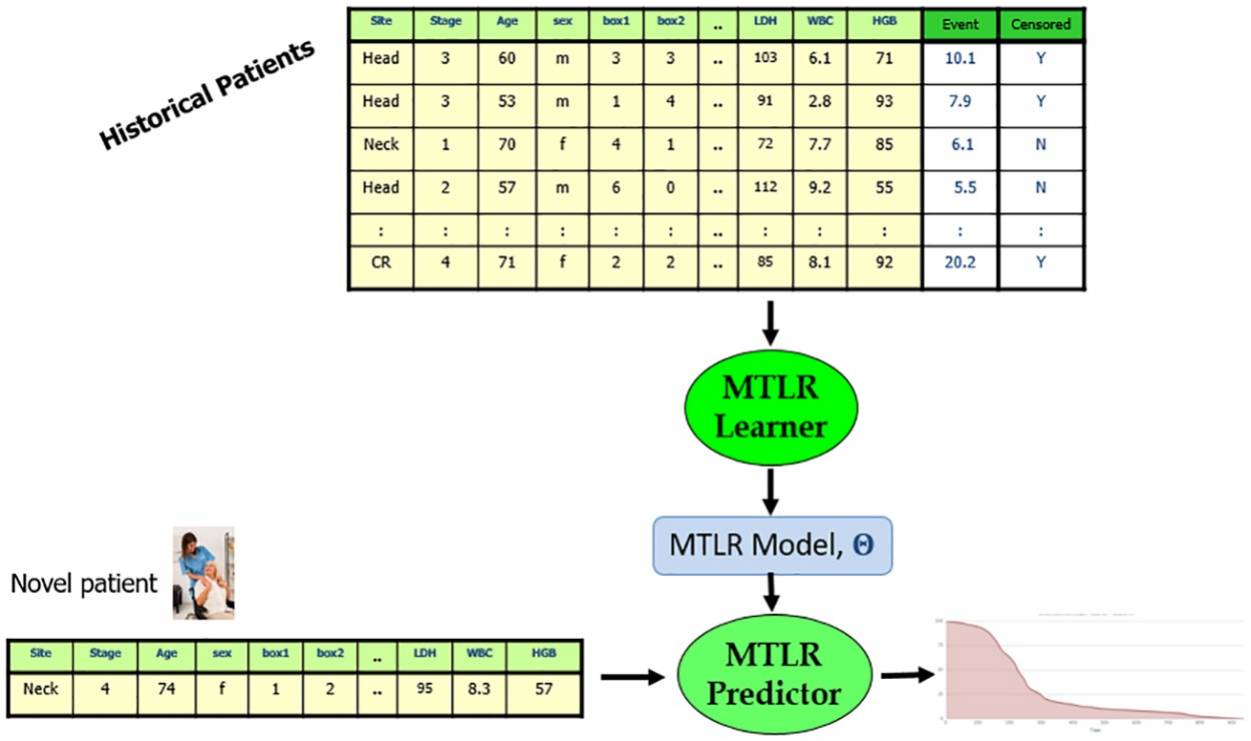
Survival prediction training and performance tasks. *Training-Task*: Historical data with event times and censor-status along with covariates are used to train a model—top-to-bottom. *Performance-Task*: new patient covariates are input to the learned model to produce a prediction of survival time—bottom, left-to-right. (Included picture designed by Freepik.).

Survival prediction is similar to regression as both involve learning a model that regresses the covariates of an individual to estimate the value of a dependent real-valued response variable—here, that variable is “time to event” (where the standard event is “death”). But survival prediction differs from the standard regression task as its response variable is not fully observed in all training instances—this tasks allows many of the instances to be “right censored”, in that we only see a *lower bound* of the response value. This might happen if a subject was alive when the study ended, meaning we only know that she lived *at least* (say) 5 years after the starting time, but do not know whether she actually lived 5 years and a day, or 30 years. This also happens if a subject drops out of a study, after say 2.3 years, and is then lost to follow-up; etc. Moreover, one cannot simply ignore such instances as it is common for many (or often, *most*) of the training instances to be right-censored; see Table 1. Such “partial label information” is problematic for standard regression techniques, which assume the label is completely specified for each training instance. Fortunately, there are survival prediction algorithms that can learn an effective model, from a cohort that includes such censored data. Each such dataset contains descriptions of a set of instances (*e.g*., patients), as well as two “labels” for each: one is the time, corresponding to the *time from diagnosis to a final date* (either death, or time of last follow-up) and the other is the *status* bit, which indicates whether the patient was alive at that final date (Fig 1).

**Table 1:** Characteristics of METABRIC and KIPAN cohorts.

	METABRIC	KIPAN
# Patients^a^	1,981	883
# Censored	1,358 (∼ 68.5%)	655 (∼ 74.4%)
# Uncensored	623	228
Time span in days (Uncensored)	3—8,941	2—5,925
# Clinical features	19	10
# Expressions (≈ #genes)^b^	49,576 (probes)	15,529
Gender	Women (100%)	Women (32.7%), Men (67.3%)

^a^ We removed the ∼50 patients from the KIPAN dataset that did not contain mRNAseq data.

^b^ While METABRIC also included copy number variations (CNV) data for the patients, here we focus on only gene expression data.

#### 2.1.1 Patient specific survival prediction using the MTLR model

This project considered 3 ways to learn a survival model: The standard approaches—Cox and Regularized Cox (RCox)—are overviewed in Appendix A.5. This subsection describes the relatively-new MTLR [14] system, which learns a model (from survival data) that, given a description of a patient **x** ∈ ℜ^*r*^, produces a *survival* curve, which specifies the probability of death *D*, *P*(*D* ≥ *t* | **x**) vs *t* for all times *t* ≥ 0. This survival curve is similar to a Kaplan–Meier curve [8], but incorporates all of the patient specific features **x**. In more detail: MTLR first identifies *m* time points {*t*_*i*_}_*i*=1‥*m*_ and then learns a variant of a logistic regression function, parameterized by *W* = {[*w*_*i*_, *b*_*i*_]}_*i*=1‥*m*_ over these *m* time points, a different such function for each time *t*_*i*_—meaning *W* is a matrix of size *m* × (*r* + 1). Using the random variable *D* for the time of death for the patient described by **x**:

$$\begin{array}{c} Pr_{W}\left(\;D\in \left[t_{k},t_{k+1}\right)\;|\;x\;\right)\;\propto \;\text{exp}(\sum_{\ell =k+1}^{m}\left({w_{\ell }}^{T}x\;+\;b_{\ell }\right)\;) \end{array}$$ (1)

**Fig 2:**
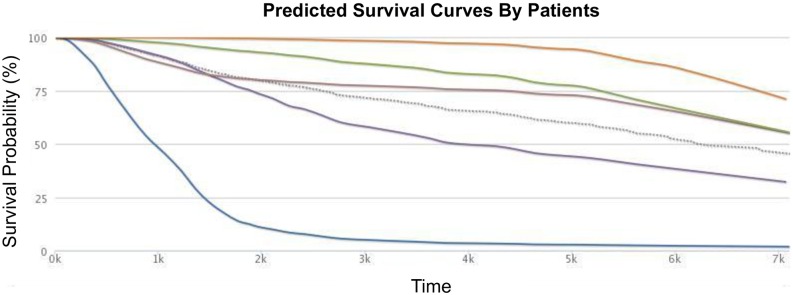
The dashed line is the Kaplan-Meier plot for this dataset. Each of the other 5 curves is a patient-specific survival curve, for 5 different METABRIC patients, from a learned MTLR model. The curves show that there are very different prognoses for the different patients, even though they all are breast cancer patients from the same cohort: here, the patient with the orange survival curve (near the top) has a very good prognosis, especially compared to the patient with the blue survival curve.

The MTLR model then combines the values of the PMF (probability mass function) into a CMF (cumulative mass function), by adding them in the reverse order—hence from the probability value of 1 at *t*_0_ = 0—*i.e*., *Pr*_*W*_(*D* ≥ 0 | **x**) = 1—down to smaller values as the time *t* increases. Fig 2 shows the individual survival curves of several patients. Here, we view a patient’s descretized survival time *d* ∈ ℜ^≥0^ as a binary vector (of classification labels) *y*(*d*) = [*y*_1_(*d*), *y*_2_(*d*), …, *y*_*m*_(*d*)], where each *y*_*j*_(*d*) ∈ {0, 1} encodes that patient’s survival status at each time interval [*t*_*j*_, *t*_*j*+1_]: *y*_*j*_(*d*) = 0 (no death yet) for all *j* with *t*_*j*_ < *d* and *y*_*j*_(*d*) = 1 (death) for all *t*_*j*_ ≥ *d*. The learning system attempts to optimize

$$\begin{array}{c} \begin{array}{c} \underset{W}{\text{min}}\;\frac{C}{2}\sum_{j=1}^{m}{\left\|w_{j}\right\|}^{2}\;-\;\sum_{i=1}^{n}[\sum_{j=1}^{m}y_{j}\left(d_{i}\right)\left(w^{T}x_{i}+b_{j}\right)-\text{log}\sum_{k=0}^{m}\text{exp}\left(f_{W}\left(x_{i},k\right)\right)]\\ \text{where}\;f_{W}\left(x_{i},k\right)\;=\;\sum_{\ell =(k+1)}^{m}\left(w_{\ell }^{T}x_{i}+b_{\ell }\right)\;\text{for}\;\;0\leq k\leq m \end{array} \end{array}$$ (2)

(This formula applies to uncensored patients; we apply the obvious extension to deal with censored instances.) This overall equation includes a L2 regularization term to reduce the risk of overfitting. The MTLR parameter *m* (the number of time points) is set to the square-root of the number of instances in all our experiments.

**Fig 3:**
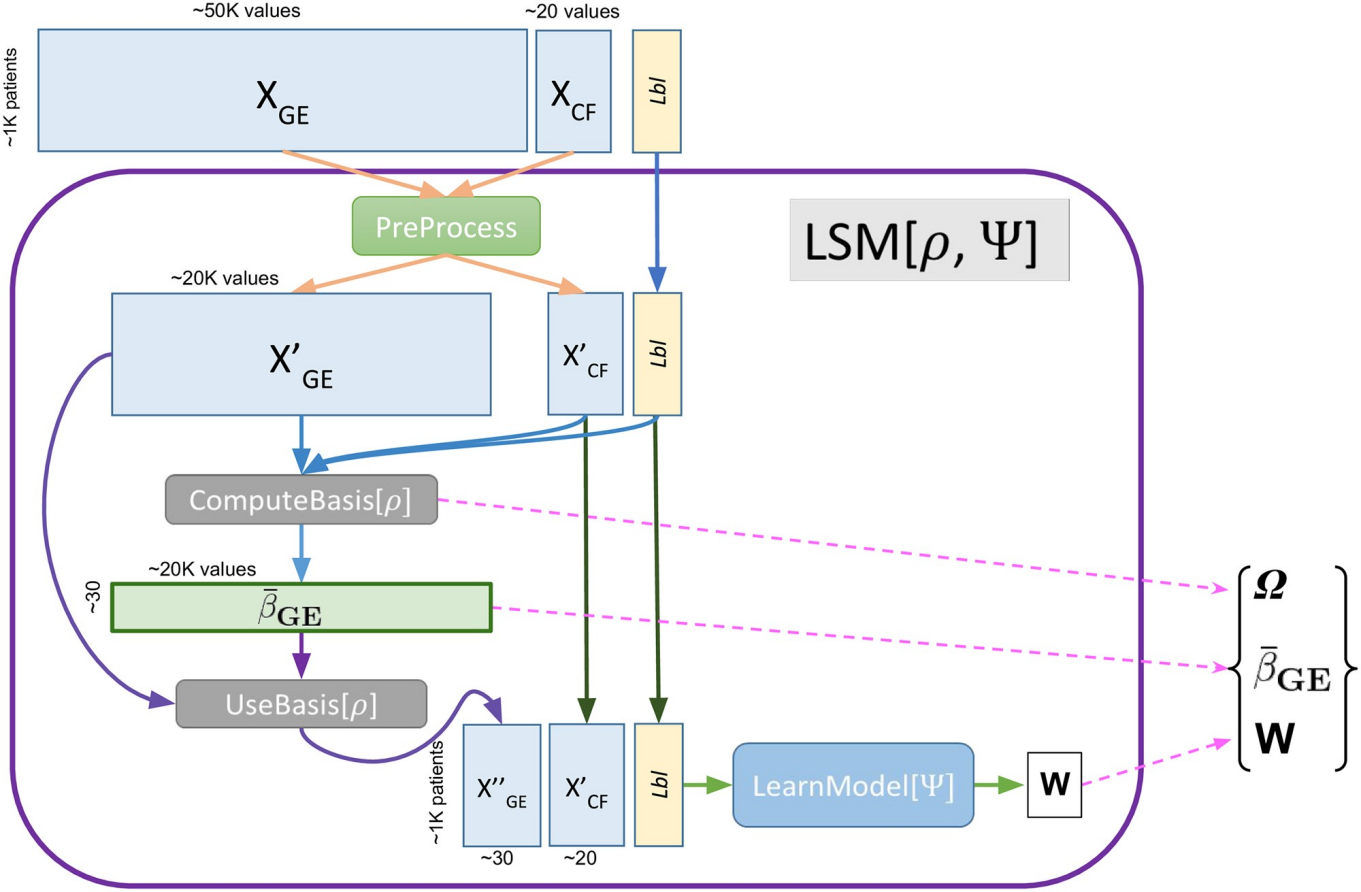
Overview of learning process. LSM = LearnSurvivalModel Uses Ψ ∈ {MTLR, Cox, RCox} for type of learner; *ρ* ∈ {dLDA, PCA} for the type of basis; *X*_*GE*_ are the gene expression values, *X*_*CF*_ are clinical features for a set of patients, “*Lbl*” is the set of their (survival prediction) labels, ${\bar{\beta }}_{GE}$ is the basis, of type *ρ* based on the instance *X*_*GE*_; *W* is the learned survival model, of type Ψ; and Ω is information about the preprocessing. Each (unrounded) box corresponds to data, whose dimensions appear around it. Each row is a patient, and each column, a feature. Each rounded box is a subroutine; here we show the input and output of each.

Given the learned parameters *W*, we can then use Eq 1 to produce a curve for each patient; we can then use the (negative of) the mean of the patient’s specific predicted survival distribution as her risk score. Yu *et al*. [14] presents more detailed explanations of model formulation, parameter learning (*W*), and the prediction task. MTLR differs from many other models (such as the standard Cox model) as: (1) MTLR produces a survival function, rather than just a risk score; and (2) MTLR does not make the proportional hazards assumption—*i.e*., it allows effect of each covariate to change with time. See also Haider *et al*. [16]. Note this is the learning process of LearnSurvivalModel (LSM_[Ψ=MTLR]_) appearing below in Fig 3, and Section 4.1.

### 2.2 Discretized Latent Dirichlet Allocation (dLDA)

Latent Dirichlet Allocation (LDA) is a widely used generative model [17], with many successful applications in natural language (NL) processing. LDA views each document as a distribution over multiple topics (document-topics distribution), where each topic is a distribution over a set of words (topic-words distribution)—that is, LDA assumes that each word in a document is generated by first sampling a topic from the document’s document-topics distribution and then sampling a word from the selected topic’s topic-words distribution. Given the set of topics (each corresponding to a specific topic-word distribution), we can view each document as its distribution over topics, which is very low dimensional.

The LDA *learning process* first identifies the latent topics—that is, the topic-words distributions corresponding to each latent topic—based on the words that frequently co-occur across multiple documents; *n.b*., it just uses the documents themselves, but not the labels. For example, it might find that many documents with the word “ball” also included “opponent” and “score”; and vice versa. Similarly, “finances”, “transaction”, and “bank” often co-occur, as do “saint”, “belief” and “pray”. Speaking loosely, the topic-model-learner might then form one topic, ${\bar{\beta }}^{1}$, that gives high probabilities to the first set of words (and relatively low probabilities to the remaining words)—perhaps

$$\begin{array}{c} \begin{array}{ccc} P_{}\left(\;\text{ball}\;|\;{\bar{\beta }}^{1}\;\right) & = & 0.05\\ P_{}\left(\;\text{opponent}\;|\;{\bar{\beta }}^{1}\;\right) & = & 0.03\\ P_{}\left(\;\text{score}\;|\;{\bar{\beta }}^{1}\;\right) & = & 0.01\\ P_{}\left(\;x\;|\;{\bar{\beta }}^{1}\;\right) & < & \text{1E-4}\;\;\text{for}\;\text{all}\;\text{other}\;\text{words}\;x \end{array} \end{array}$$

(Technically, each topic is specified as a Dirichlet distribution over the set of words, *β*^*i*^. To simplify the presentation, here we are showing their expected values, these ${\bar{\beta }}^{i}=E\left[\beta^{i}\right]$ values are based on the priors; see Appendix A.2(3).)

This ${\bar{\beta }}^{1}$ corresponds to an *n*-tuple over the *n* words; we call this ${\bar{\beta }}^{1}\approx \left[P_{}\left(\;w_{1}\;|\;{\bar{\beta }}^{1}\;\right),\;\ldots ,\;P_{}\left(\;w_{n}\;|\;{\bar{\beta }}^{1}\;\right)\right]$. It would similarly identify a second topic ${\bar{\beta }}^{2}$ with the *n*-tuple ${\bar{\beta }}^{2}\approx \left[P_{}\left(\;w_{1}\;|\;{\bar{\beta }}^{2}\;\right),\;\ldots ,\;P_{}\left(\;w_{n}\;|\;{\bar{\beta }}^{2}\;\right)\right]$ that gives high probabilities to the different set of words, etc. (While we might view the first topic as related to sports, the second related to finances, and third to religion, that is simply our interpretation, and is not needed by the learning algorithm. Other topics might not be so obvious to interpret.) This produces the *topic-words distribution* $B\;=\;{\left\{{\bar{\beta }}^{i}\right\}}_{i=1‥K}$ over *K* topics.

The learner would then map each document into a “distribution” over this set of *K* topics—perhaps document *f*_1_ would be decomposed as Θ(*f*_1_) = [*θ*_1_(*f*_1_), *θ*_2_(*f*_1_) …, *θ*_*K*_(*f*_1_)] = [0.01, 13.02, 50.01 …, 0.03]—these are parameters for a Dirichlet distribution, which are non-negative, but do not add up to 1. These are different for different documents—*e.g*., perhaps *f*_2_ is expressed as Θ(*f*_2_) = [12.03, 0.001, 3.1, …, 2.4], etc. This is the *document-topic distribution* {Θ(*f*_*j*_)}_*j*=1‥*m*_ over the *m* documents.

The specific learning process depends on the distributional form of the document-topics and topic-words distributions (here, we use Dirichlet for both) and also the number of latent topics, *K*. Given this, the LDA learning process finds the inherent structure present in the data—*i.e*., a model (topic-words distributions for each of the *K* topics ${\left\{{\bar{\beta }}^{i}\right\}}_{i=1‥K}$) that maximizes the likelihood of the training data.

The same way certain sets of words often co-occur in a document, similarly sets of genes are known to be co-regulated: under some condition (corresponding to a “c_topic”), every gene in that set will have some additional regulation—some will be over-expressed, each by its own amount, and the others will be under-expressed. Moreover, just as a natural language (NL) topic typically involves relatively few words, most c_topics effectively involve relatively few genes. Also, just like a document may involve a mixture of many topics, each to its own degree, so a patient’s cancer often involves multiple c_topics; see work on cancer subclones [18]. This has motivated many researchers to use some version of topic modeling to model gene expression values, under various (sets of) conditions.

For example, Rogers *et al*. [19] proposed Latent Process Decomposition (LPD), a probabilistic graphical model that was inspired by LDA, for microarray data, and presented clustering of genes that led to results comparable to those produced by hierarchical clustering. (Their results are descriptive; they do not use the results in any downstream evaluation). Later Masada *et al*. [20] proposed improvements to the original LPD approach and showed similar results. Bicego *et al*. [21] report topic modeling approaches (including LPD) were useful in classification tasks with gene expression data. They applied several topic models as dimensionality reduction tools to 10 different gene expression data sets, and found that the features from the topic models led to better predictors.

Further, Lin *et al*. [22] reviewed various different topic models applied to gene expression data, including LDA and probabilistic latent semantic analysis (PLSA) [23], as well as the topic model approaches described above, for gene classification and clustering. They note that the topic model approaches improve over other models as one can easily interpret the topic-words distributions and the mixed membership nature of the document-topic distribution.

However, none of these tasks were survival analysis. They used shifting and scaling to convert the continuous gene expression values to discrete values; we considered this approach for our data, but found that it was not able to learn distinct topics for our data. Moreover, this gave all patients very similar document-topic distributions. Dawson *et al*. [24] proposed a survival supervised LDA model, called survLDA, as an extension of supervised LDA [25]. survLDA uses a Cox model [26] to model the response variable (survival time) instead of the generalized linear model [27] used in supervised LDA [25]. But Dawson *et al*. [24] reported that the topics learned from survLDA were very similar to the ones learned from the general (unsupervised) LDA model.

**Fig 4:**
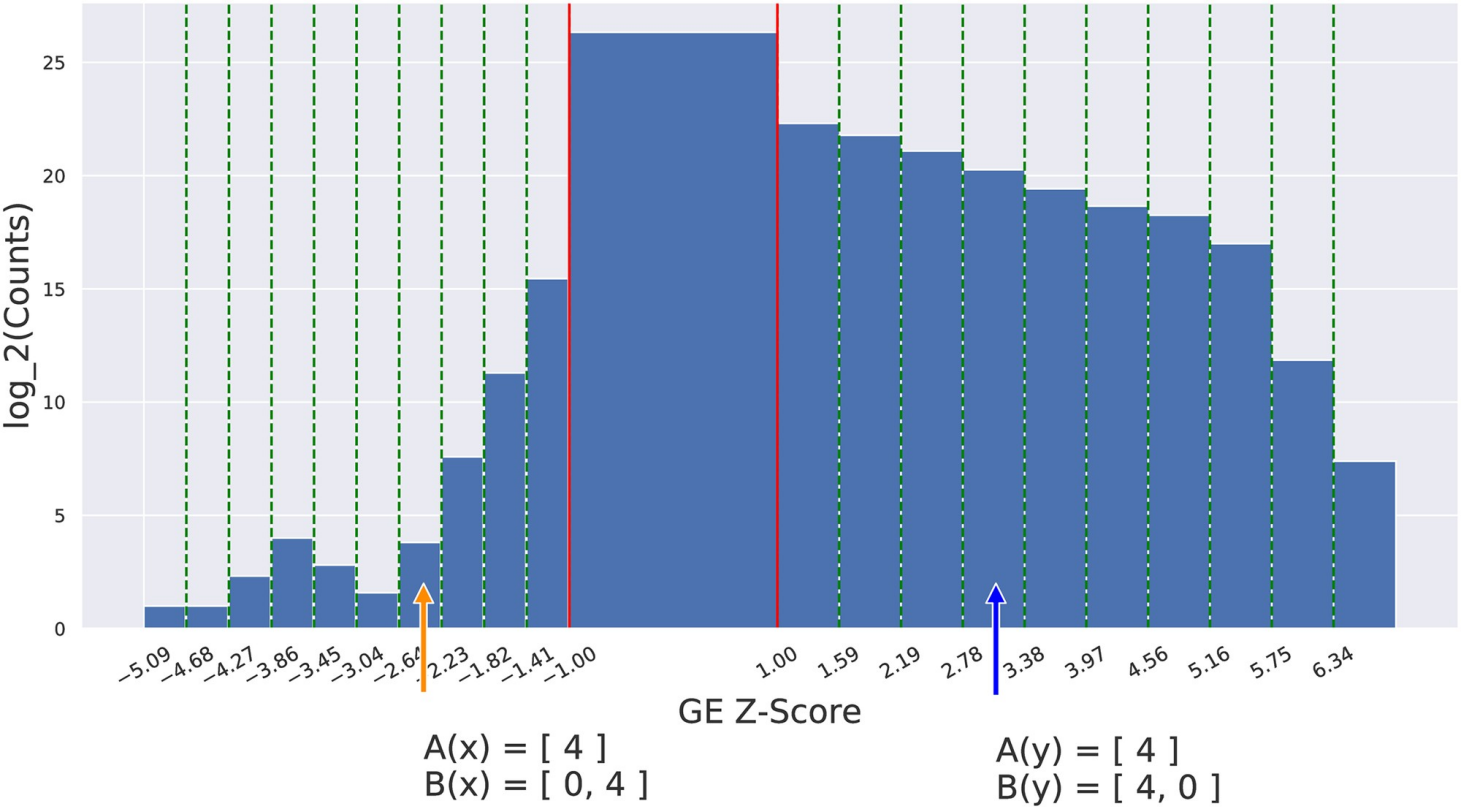
Histogram of the normalized Gene Expression values $\left\{x_{i}^{j}\right\}$, from METABRIC, showing how we descretized them into essentially equal-width bins. Note the heights are on a log-scale. The material under the histogram—involving **x** and **y**—compare two ways to compute the discretized Gene Expression Values (dGEV): The top Enc_A discretizes the GEVs into a single count-feature A(⋅), and the bottom Enc_B discretizes the GEVs into two count-features B(⋅) representing over-expression and under-expression, respectively.

Here, we apply the “standard” topic-modeling approach to gene expression data, for the survival prediction task. While previous systems applied topic modeling techniques to gene expression data, very few have applied topic models to predict a patient’s survival times (and none to our knowledge have used mRNAseq expression data). Our work presents a more direct analogue to the NL topic modeling that can be applied to our cohort of patients with gene expression data, where each patient corresponds to a document and the genes/probes in the expression data correspond to the words that form the document. This requires making some significant modifications to the standard LDA model, which assumes the observations are frequencies of words, which are non-negative integers that generally follows a monotonically decreasing distribution. By contrast, gene expression values are arbitrary real values, believed to follow a skewed Gaussian distribution [28]; see also Fig 4. (This is also true for mRNAseq, as we need to normalize the expression counts to be comparable, from patient to patient).

**Fig 5:**
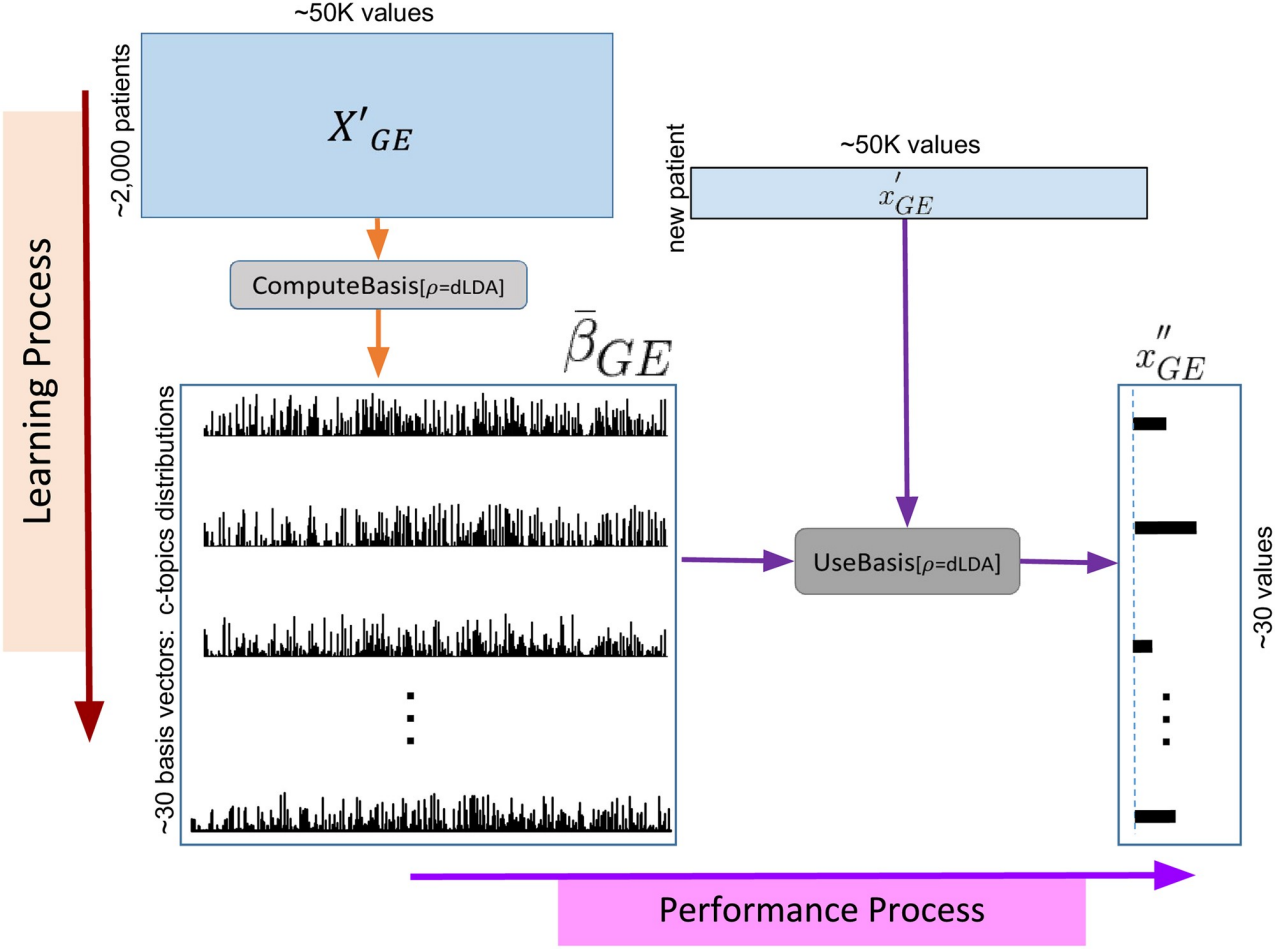
The ComputeBasis_[*ρ*=dLDA]_ process (shown top-to-bottom, on left) uses a set of high-dimensional gene expression vectors $X_{GE}^{\prime }$ from many patients, to produce a set of basis vectors (corresponding the parameters of the LDA) ${\bar{\beta }}_{GE}$. The UseBasis_[*ρ*=dLDA]_ process (shown left-to-right horizontally) uses the set of dLDA “basis vectors” ${\bar{\beta }}_{GE}$ to transform a gene expression vector $x_{GE}^{\prime }$ from a novel patient, into a low-dimensional description $x_{GE}^{\prime \prime }$. (Note ComputeBasis_[*ρ*=dLDA]_ also uses other information, $X_{CF}^{\prime }$ and *Lbl*, to determine the number *K* of basis vectors; to simplify the figure, we did not show this.).

We follow the approach of explicitly discretizing the expression values in a preprocessing step, so the resulting values basically, approximate a Zipf distribution. There are still some subtleties here—*e.g*., while the NL situation involves only non-negative integers, an affected gene can be either over-expressed, or under-expressed—*i.e*., we need to deal with two directions of “deviation”, while NL’s LDA just deals with one direction; see Section 4.1.1. We refer to our model as *dLDA* and the discretized gene expression values as *dGEVs*. The same way the standard LDA approach reduces the description of a document from a ≈ 10^5^-dimensional vector (corresponding to the words used in that document) to a few dozen values (the “distribution” of the topics), this dLDA approach reduces the ≈ 50*K*-dimensional gene expression tuple to a few dozen values—here the “distribution” of the c_topics. Fig 5 summarizes this process: using the subroutines defined in Section 4 below, at learning time, ComputeBasis_[*ρ*=dLDA]_ first identifies the set of relevant c_topics ${\bar{\beta }}_{GE}$ from the set of gene expression values $X_{GE}^{\prime }$, then later (at performance time), UseBasis_[*ρ*=dLDA]_ uses those learned c_topics to transform a new patient’s high-dimensional gene expression profile $x_{GE}^{\prime }$ to a low-dimensional c_topic-profile, *x*”_*GE*_—here going from 50K values to 30.

Section 5 presents empirical evidence that this method works effectively for our survival prediction task; Appendix C.1 shows that it performs better than the LPD technique.

## 3 Datasets used

We apply our methods to two large gene expression datasets: the METABRIC breast cancer cohort [7] (mircroarray) and the Pan-kidney cohort KIPAN (mRNAseq) [29, 30]. We initially focus on the METABRIC dataset [40] which is one of the largest available survival studies that includes genomic information. In 2012, the Breast Cancer Prognostic Challenge (BCC) organizers released the METABRIC (Molecular Taxonomy of Breast Cancer International Consortium) dataset for training [7]. While they subsequently released a second dataset (OSLO) for final testing [7], we are not using it for several reasons: (1) METABRIC provided disease-specific survival (DS), which considers only *breast cancer death* (BC-based death), rather than all causes of death [13]. By contrast, OSLO provides “overall survival”, which does not distinguish BC-based deaths from others. As DS is clearly better for our purpose, it is better to evaluate on the METABRIC dataset. (2a) OSLO and METABRIC contained different sets of probes—and in particular, OSLO contains only ∼ 80% of the METABRIC probes. (2b) Similarly, the OSLO dataset is also missing some of the clinical covariates that are present in the METABRIC dataset—*e.g*., menopausal status, group, stage, lymph nodes removed, etc.; see [3, Table 1]. This means a “METABRIC-OSLO study” would need to exclude some METABRIC features and some METABRIC probes.

We then used a second independent dataset, to verify the effectiveness of our “dLDA+MTLR” approach. Here, we did not use OSLO, as we wanted to explore a different type of cancer, and also use a different platform, to show that our system could still identify an appropriate (and necessarily different) set of cancer-topics (c_topics). We therefore used the KIPAN dataset from TCGA (The Cancer Genome Atlas), as it (also) contains a large number of patients and provides survival information.

Table 1 lists some of the important characteristics of these datasets. Note that KIPAN contains 15,529 genes, while METABRIC has 49,576 probes. This is because many METABRIC probes may correspond to the same gene each targeting a different DNA segment of the gene. As different probes for the same gene might behave differently, we gave our learning algorithm the complete set of probes. Our results on the KIPAN dataset show that our approach also works when dealing with gene expression data from a totally different cancer and platform (here kidney not breast, and mRNAseq rather than Microarray)—demonstrating the generality of our approach.

### 3.1 Training vs test data

We apply the same experimental procedure to both datasets (METABRIC and KIPAN): We partition each dataset into two subsets, and use 80% of the data for training and the remaining 20% for testing. Both partitions contain instances with comparable ranges of survival times and comparable censored-versus-uncensored ratio. When necessary, we ran internal cross-validation, within the training set, to find good settings for parameters, etc.

## 4 Overview of learning and performance processes

**Fig 6:**
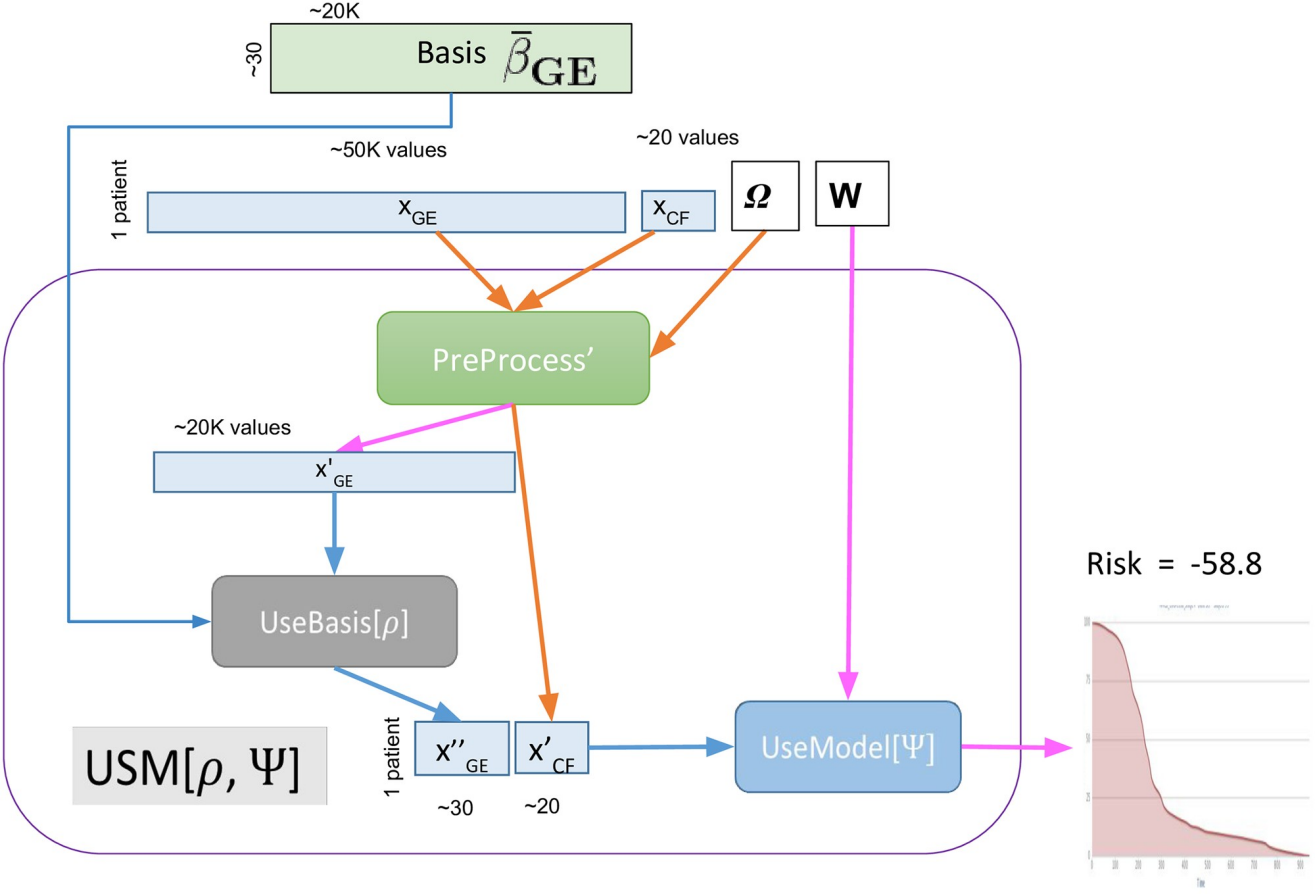
Overview of performance process, USM = UseSurvivalModel. *x*_*GE*_ and *x*_*CF*_ are the gene expression values and clinical feature values, for a single patient; see also terms *ρ*, Ψ, Ω, ${\bar{\beta }}_{GE}$, *W* from Fig 3.

As typical for Supervised Machine Learning systems, we need to define two processes:

- The learning algorithm, LearnSurvivalModel
  LSM_[*ρ*=dLDA; Ψ=MTLR]_([*X*_*GE*_, *X*_*CF*_], *Lbl*) takes a labeled dataset, involving both gene expression data *X*_*GE*_ and clinical features *X*_*CF*_ (and survival-labels *Lbl*) for many patients, and computes a Ψ = MTLR survival model *W*. (Many subroutines are parameterized by a dimensionality reduction technique *ρ* ∈ {dLDA, PCA}, and/or by a survival learning algorithm Ψ ∈ {MTLR, Cox, RCox}. We use notation “Alg_[*ρ*; Ψ]_(⋅)” to identify the specific parameters; hence LSM_[*ρ*=dLDA; Ψ=MTLR]_([*X*_*GE*_, *X*_*CF*_], *Lbl*) is dealing with the *ρ* = dLDA encoding and Ψ = MTLR survival learning algorithm).
  It also returns the *ρ* = dLDA “basis set” ${\bar{\beta }}_{GE}$ (here, think of a set of c_topic distributions), and some information about the pre-processing performed, Ω. See Fig 3.
- The performance algorithm, UseSurvivalModel
  USM_[*ρ*=dLDA; Ψ=MTLR]_([*x*_*GE*_, *x*_*CF*_], ${\bar{\beta }}_{GE}$, *W*, Ω), takes a description of an individual (both gene expression *x*_*GE*_, and clinical features *x*_*CF*_), as well as the *ρ* = dLDA basis set ${\bar{\beta }}_{GE}$ and the Ψ = MTLR survival model *W* (and pre-processing information Ω), and returns a specific survival prediction for this individual, from which we can compute that person’s risk score. See Fig 6.

To simplify the presentation, the main text will describe the process at a high-level, skipping most of the details. Notice these functions are parameterized by the type of dimensionality reduction *ρ* and the survival learner Ψ. This section will especially focus on the novel aspects here, which are the *ρ* = dLDA transformation (Section 2.2), which complicates the ComputeBasis_[*ρ*=dLDA]_(⋯) function (Section 4.1.1); and the Ψ = MTLR algorithm for learning the survival model (Section 2.1.1). Appendix A summarizes the more standard *ρ* = PCA approach to reducing the number of features, and the more standard survival models Ψ ∈ {Cox, RCox}, as well as other details about the learning, and performance models, in general.

### 4.1 Learning system LSM

Here, LSM_[*ρ*=dLDA; Ψ=MTLR]_ ([*X*_*GE*_, *X*_*CF*_], *Lbl*) first calls PreProcess, which fills-in the missing values in the *X*_*CF*_ clinical features (producing $X_{CF}^{\prime }$), and normalizes the real-valued *X*_*GE*_ genetic features, which is basically computing the z-scores $X_{GE}^{\prime }$, over *all* of the values. It then calls ComputeBasis_[*ρ*=dLDA]_(⋯) to compute a set of c_topics ${\bar{\beta }}_{GE}$ from the gene expression data $X_{GE}^{\prime }$ (as well as the other inputs), then calls UseBasis[*ρ* = dLDA]($X_{GE}^{\prime },\;{\bar{\beta }}_{GE}$), which “projects” $X_{GE}^{\prime }$ onto this ${\bar{\beta }}_{GE}$ to find a low dimensional description of the genetic information; see Fig 5. These projected values, together with $X_{CF}^{\prime }$ and *Lbl*, form the labeled training set given to the Ψ = MTLR learning system, which computes a survival model *W*. Here, the LSM process returns the dLDA “basis” ${\bar{\beta }}_{GE}$ and the MTLR-model *W*. (Further details appear in Appendix A.)

#### 4.1.1 ComputeBasis_[*ρ*=dLDA]_(⋯) function

As noted above, the ≈50,000 expression values for each patient is so large that most standard learning algorithms would overfit. We consider two ways to reduce the dimensionality. One standard approach, Principal Component Analysis (PCA), is discussed in Appendix A.4. Here, we discuss a different approach, dLDA, that uses the Latent Dirichlet Analysis.

The PreProcess routine computes z-scores $X_{GE}^{\prime }$ for the gene expression values *X*_*GE*_; the ComputeBasis_[*ρ*=dLDA]_ subroutine then has to transform those real values to the non-negative integers required by LDA—moreover, it was designed to deal with word counts in documents where, in any given document, most words appear 0 times, then many fewer words appear once, then yet fewer words appear twice, etc. We therefore need a method for converting the real values into non-negative integers.

This process therefore *discretizes* the standardized gene expression values (in $X_{GE}^{\prime }$) into the integers {-10, -9, …, -1, 0, 1, …, 9, 10}, by mapping each real number to the integer indexing some essentially equal-sized bins; see Fig 4, and Appendix A.2 for details.

This does map each gene expression to an integer, but this includes both positive and negative values. Given that over-expression is different from under-expression, an obvious encoding uses two non-negative integer values for each gene: mapping +2 to [2, 0], and −3 to [0, 3], etc. Note that the range of each component of the encoding will be non-negative integers, and that most of the values will be 0, then fewer will be 1, etc.—as desired. However, this does double the dimensionality of representation; *i.e*., we now have twice the number of genes: UNDER-‘gene_name’ and OVER-‘gene_name’.

(Below we call this the Enc_B encoding; see also *B*(⋅) at the bottom of Fig 4.) Given that very few values are < −1 (in METABRIC, over 14% (normalized) expression values were > 1, but less than less than 0.04% were < −1; recall that heights in Fig 4 are on a log scale), we considered another option: collapsing the +values and −values to a single value—so both +4 and −4 would be encoded as 4. This would mean only half as many features (which would reduce the chance of overfitting), and would continue to note when a gene had an exceptional value. (This is the Enc_A encoding, which corresponds to the *A*(⋅) at the bottom of Fig 4.) As it was not clear which approach would work better, our implementation explicitly considered both options—and used the training set to decide which worked best; see below.

The standard LDA algorithm also needs to know the number of topics (here c_topics) *K* to produce. ComputeBasis uses (internal) cross-validation to find the best value for *K*, over the range *K* ∈ {5, 10, 15, …, 150}, as well as encoding technique *t* ∈ {Enc_A, Enc_B}—seeking the setting leading to the Cox model with the best concordance (on each held-out portion). See Appendix A.2 for details. After finding the best K* and encoding t*, ComputeBasis then finds the K* c_topics on the t*-encoded (preprocessed) training gene expression data $X_{GE}^{\prime }$; this is the c_topic distribution, ${\bar{\beta }}_{GE}$.

The vertical left-side of Fig 5 gives a high-level description of the ComputeBasis_[*ρ*=dLDA]_ process: given a large set of (preprocessed) high-dimensional gene expression profiles, produce a small set of c_topics (each corresponding to a mapping from the gene expression profiles). We will later describe the UseBasis_[*ρ*=dLDA]_ process that uses those c_topics to transform the high-dimensional gene expression profile of a novel instance, into a small dimensional set of values—see the left-to-right “Performance Process” part here. At this abstract level, it is easy to see that it nicely matches the *ρ* = PCA process, where ComputeBasis_[*ρ*=PCA]_ would find the top principle components of the $X_{GE}^{\prime }$ datasets (here, the ${\bar{\beta }}_{GE}$ box would be those components), which UseBasis_[*ρ*=PCA]_ could then use to transform a new gene expression profile into that low-dimensional “PC-space”.

### 4.2 Performance system, USM

As shown in Fig 6, the USM_[*ρ*=dLDA;Ψ=MTLR]_([*x*_*GE*_, *x*_*CF*_], ${\bar{\beta }}_{GE}$ *W*, Ω) system applies the learned Ψ = MTLR model *W*, to a PreProcess’ed description of a novel patient, $\left[x_{GE}^{\prime \prime },x_{CF}^{\prime }\right]$, whose gene expression values $x_{GE}^{\prime \prime }$ have been “projected” into the relevant basis ${\bar{\beta }}_{GE}$ by UseBasis_[*ρ*=dLDA]_. This produces a survival curve, which it then uses to produce that patient’s predicted risk score: the negative of the expected time for this distribution, which corresponds to the area under its survival curve.

Each of the various subroutines are described in an appendix: PreProcess’, UseBasis_[*ρ*=dLDA]_ and UseBasis_[*ρ*=PCA]_ are described in Appendices A.1, A.3 and A.4, respectively. The UseModel_[Ψ=Cox]_ and UseModel_[Ψ=RCox]_ produce standard risk scores for each patient, obtained by applying the learned Cox (resp., RCox) model to the patient’s clinical and gene expression features; see Appendix A.5.

## 5 Experimental results

As noted above, we intentionally designed our learning and performance systems (Figs 3 and 6) to be very general—to allow two types of basis *ρ* ∈ {dLDA, PCA} and three different survival prediction algorithms Ψ ∈ {MTLR, Cox, RCox}. This allows us to explore 2 × 3 frameworks, on the two different datasets (METABRIC and KIPAN). For each, the learner uses internal internal cross-validation to find the optimal parameters. Below we report the results of each optimized model on the held-out set, focusing on the Concordance Index (CI)—a discriminator measure. We also discuss a *calibration* measure of these results; see Appendix B.2.

We also present our experimental results from the BCC Dream Challenge winner’s model [13]. As discussed in Section 4.1.1, ComputeBasis_[*ρ*=dLDA]_ ran internal cross-validation on the training set to determine the appropriate encoding t* ∈ {Enc_A, Enc_B} and the optimal number of c_topics for the dLDA model K* from a large potential values (see Algorithm 1 in Appendix A.2). Our experiments found that the discretization t* = Enc_B, along with K* = 30 c_topics, produced the best dLDA algorithm for survival prediction in METABRIC; after fixing the encoding scheme as Enc_B, we used the same technique on the KIPAN dataset and found *K* = 50 c_topics to be the best. We used the C implementation from Blei *et al*. [17, 41] to compute the c_topics. On a single 2.66GHz processor with on 16Gb memory, a single fold takes around ∼20–30 hours (more time for larger *K*). We, of course, parallelized each CV fold.

We experimented with different combinations of the features from three groups: (1) clinical features, (2) SuperPC+ principle components (*ρ* = PCA), and/or (3) the dLDA c_topic (*ρ* = dLDA); and with three different survival prediction algorithms Ψ ∈ {Cox, Cox, MTLR}. Our goal in these experiments is to empirically evaluate the performance of the survival models that use various types of features. Given this goal, we evaluate the performance using different GE basis methods (*ρ*) by comparing their performance to a baseline model that only uses the clinical features with Cox [26]. The other combinations include clinical features as well as various different GE features; each is trained using each of the three aforementioned survival prediction algorithms (Ψ).

As an additional feature selection step, we removed the covariate “Site” from the METABRIC clinical covariates, based on our experimental results (on the training data) that shows its inclusion led to worse concordance. We experimented with a large, but selective, set of model combinations, to answer our major queries:

(i) does adding GE features improve survival prediction?
(ii) which is the best feature combination for survival prediction?
(iii) which is better representation of the GE features: dLDA or SuperPC+?
(iv) are we deriving GE features that are redundant with PAM50?

**Fig 7:**
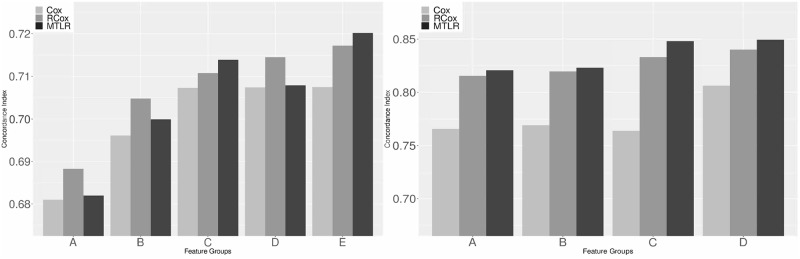
Test CI: METABRIC (left) and KIPAN (right). Note higher values are better. The labels on the x-axis correspond to Table 2.

Our results appear in Table 2, shown visually in Fig 7(left). Note the baseline is “A-Cox”, where the ‘A’ refers to the feature set used, which here is the far left triplet of blocks in Fig 7(left), and the ‘Cox’ refers to the learning algorithm, which appears left-most in each triplet. These results lead us to claim:

(i) Comparing the baseline, A-Cox, to the other models, we immediately see that adding GE features (using any of the dimensionality reduction technique) leads to better predictive models;—*i.e*., all of the results are better than A-Cox’s CI of 0.6810 (the left-most light-shaded bar in Fig 7(left)).
(ii) The best model for METABRIC is the one that includes all of the types of features derived from the gene expression—here E-MTLR, which is the right-most bar of Fig 7(left).
  We also performed student’s t-tests on random bootstrap samples from the test data to validate the significance of our results. When we compare this best model, E-MTLR, against models B-RCox (which is the best model using only PCA GE features) and C-MTLR (the best model using only dLDA GE features), we find statistically significant difference between them (respective pairwise p-value: 4.8e-16, 1e-3), showing that the E-MTLR model is significantly better than its closest counterparts.
(iii) These empirical results show that, if you are pick only a single GE feature set, the dLDA c_topics perform better than the principle components—that is, the C-*χ* has a higher score than B-*χ*, for *χ* ∈ {Cox, RCox, MTLR}; moreover, a model using both sets of features performs yet better (*i.e*., D-*χ* is better that C-*χ*).
(iv) Comparing the D-*χ* to E-*χ*, we see that adding PAM50 subtypes as features to the METABRIC database improves the held-out test concordance.

**Table 2:** Concordance results of various models from METABRIC (over 395 test instances) and KIPAN (over 176 test instances). + = used these features and − = did not use these features As PAM50 is specific to breast cancer, it is *not applicable* to the kidney (KIPAN) data. The first row, with the ID “A(*)”, is the baseline.

ID	Feature Groups				Learning Alg.	Concordance	
	Clinical	PCA	dLDA	PAM50	Cox | RCox | MTLR	METABRIC	KIPAN
A (*)	+	−	−	−	Cox	0.6810	0.7656
A	+	−	−	−	RCox	0.6883	0.8156
A	+	−	−	−	MTLR	0.6820	0.8207
B	+	+	−	−	Cox	0.6961	0.7691
B	+	+	−	−	RCox	0.7048	0.8196
B	+	+	−	−	MTLR	0.6999	0.8232
C	+	−	+	−	Cox	0.7073	0.7638
C	+	−	+	−	RCox	0.7108	0.8332
C	+	−	+	−	MTLR	0.7139	0.8482
D	+	+	+	−	Cox	0.7074	0.8062
D	+	+	+	−	RCox	0.7145	0.8401
D	+	+	+	−	MTLR	0.7079	**0.8495**
E	+	+	+	+	Cox	0.7075	NA
E	+	+	+	+	RCox	0.7172	NA
E	+	+	+	+	MTLR	**0.7202**	NA
F	+	Meta-Genes			Ensemble Model	0.7293	NA

Indeed, we see that the performance of models that include PAM50 are marginally better than similar models that do not (row D), suggesting that the information added by these different representations of GE data are not redundant. Moreover, we see that, in all feature groups, both RCox and MTLR clearly outperform Cox—*i.e*., *ν*-RCox and *ν*-MTLR are better than *ν*-Cox, for *ν* ∈ {A,B,C,D,E}. We then tested the first three claims on the KIPAN dataset; see Table 2 (right-most column) and Fig 7(right). (As KIPAN does not deal with breast cancer, the PAM50 features are not relevant, so we could not test claim (iv).)

(i) As before, we found that adding expression information improves over the baseline A-Cox –*i.e*., essentially all values are better than 0.7656.
(ii) We again found that the best model was the one that included all of the features; here D-MTLR. Moreover, a t-test on bootstrap replicas show that this model D-MTLR was *significantly* better than the top model that does not include dLDA features, B-MTLR.
(iii) We again see that C-*χ* has a higher score than B-*χ*, meaning (again) that models trained with *only the c_topics performed much better than PCA-features*; but that including both features was yet better (D-*χ*).

These sets of experiments support our claim that

a model learned by running MTLR on all GE features, gives very good concordance scores

–statistically better than other options in two different datasets, using different platforms, related to different cancer types.

In addition to these evaluations using the discriminative concordance measure, we also applied a calibration measure: “D-Calibration” (“D” for “Distribution”) [15, 16], which measures how well a individual survival distribution model is calibrated, using the Hosmer-Lemeshow (HL) [30] goodness-of-fit test; see Appendix B.2. We found that all of our models, for both datasets (METABRIC and KIPAN), passed this calibration test; see Appendix C.2, especially Table 3. But we have found that this is not universal. For example, we experimented with another breast cancer dataset BRCA (results not shown here), and found that the Cox model failed for all configurations (of *ρ*), showing that the Cox model does not always produce calibrated results—here, for situations where RCox and MTLR produced D-calibrated predictors. See also Haider *et al*. [16].

**Table 3:** D-calibration results from METABRIC, KIPAN, and BRCA, on the held-out test data. p-values greater than 0.05 suggest the model is good (“D-calibrated”). Note that Cox models fail D-calibration test for all feature combinations, for BRCA dataset.

ID	Feature Groups			Algorithms	KIPAN		METABRIC		BRCA	
	Clinical	PCA	dLDA		HL-Statistic	p-value	HL-Statistic	p-value	HL-Statistic	p-value
A	+	−	−	Cox	7.1455	0.9888	12.6765	0.8104	52.5289	**3.1e-05**
A	+	−	−	RCox	7.1279	0.9890	14.8143	0.6747	4.8816	0.9990
A	+	−	−	MTLR	8.2421	0.9748	11.2300	0.8843	6.6300	0.9929
B	+	+	−	Cox	5.6697	0.9973	8.1723	0.9760	63.7983	**4.9e-07**
B	+	+	−	RCox	2.5769	0.9973	6.4499	0.9940	6.0013	0.9962
B	+	+	−	MTLR	8.4315	0.9714	10.8421	0.9009	8.9895	0.9600
C	+	−	+	Cox	10.54166	0.9127	6.0066	0.9962	48.9904	**0.0001**
C	+	−	+	RCox	7.9878	0.9993	4.1344	0.9997	4.6031	0.9993
C	+	−	+	MTLR	6.5578	0.9933	8.2000	0.9755	9.2421	0.9539
D	+	+	+	Cox	14.1951	0.7162	11.2333	0.8842	25.2241	**0.1189**
D	+	+	+	RCox	8.2420	0.9748	3.4981	0.9999	6.0813	0.9959
D	+	+	+	MTLR	7.4947	0.9852	6.5158	0.9936	9.0211	0.9593

### 5.1 Other comparisons

In 2012, Cheng *et al*. [13] won the BCC Dream Challenge (which was based on the METABRIC data) by (i) leveraging prior knowledge of cancer biology to form Meta-Genes and (ii) training an ensemble of multiple learners, fueled by the continuous insights from the challenge competitors via open sharing of code and trained models. To compare our performances with this BCC winning program, we reproduced their models (using the DreamBox7 package), then re-trained their ensemble learners on our training split of the METABRIC data and tested on the held-out test set. Table 2[Row *F*] shows that the resulting ensemble model achieved a CI of 0.7293 on the test data. While that score is slightly better than the performance of our best model (Table 2[Row *E*]), note that all of our tuning was performed solely on the training (n = 1586) data, while their team made major design choices for their model using the entire METABRIC cohort (all n = 1981 instances), on which it was then evaluated.

Recently, Yousefi *et al*. [31] trained a deep neural network on this KIPAN data—including this gene expression data, as well as other features: Mutation, CNV and Protein. They reported concordance scores around 0.73−0.79, which are lower than our best, 0.8495.

Finally, while we focused on the LDA approach, we also explored another topic modeling technique, Latent Semantic Indexing (LSI) [23]. Running this on both datasets (using the same discretization approach, the same t* = Enc_B encoding and the same number of c_topics, K* = 30), we found essentially the same Concordance values, and confirmed that all four claims (i) through (iv) still hold, just replacing dLDA with the “discretized LSI” (dLSI) encoding.

## 6 Discussion

Given the growing number of gene expression datasets as part of survival analysis studies, it is clearly important to develop survival prediction models that can utilize such high-dimensional GE data. This motivated us to propose a novel survival prediction methodology that can learn predictive features from such GE data—exploring ways to learn and use c_topics as features for models that can effectively predict survival. *N.b*., this paper focuses exclusively on this predictive task, as this can lead to clinically relevant patient-specific information; indeed, this motivated the BCC Dream challenge, which provided the METABRIC dataset. We anticipate future work will explore the possible interpretation of these c_topics.

We included Cox as one of our learning modules for this task as it is known to be effective at optimizing concordance, both empirically and theoretically [32]. We included RCox as this algorithm recently won Prostate Cancer Dream Challenge 9.5 [12]. Finally, we included the MTLR survival prediction model as its performance, there, was competitive with the best, as well as based on the empirical evidence in Haider *et al*. [16]. Our evaluations on these two datasets show that MTLR’s performance was often better than RCox and Cox. Moreover, while the basic RCox and Cox functions produce only a risk score for each patient, MTLR provides a survival distribution for each, mapping each time to a probability; see Fig 2. Such models, which produce an individual survival distribution, can be used to compute a risk score, allowing them to be used for concordance-tasks; they can also be used to predict single time probabilities (*e.g*., probability of a patient living at least 3 years), and also can be visualized. (We used the Kalbfleisch-Prentice approach to estimate the base hazard function, allowing Cox and RCox to similarly produce individual survival curves. Appendix B.2 describes a way to evaluate such “individual survival distribution” models, D-Calibration. Appendix C.2 then shows that, for these datasets, these models all pass this test).

To summarize the main disadvantages and advantages of our approach, versus more standard approaches (*e.g*., PCA for dimensionality reduction, and (R)Cox for survival prediction):

- Disadvantages:
  - Topic models are not simple to describe.
  - This approach requires a fairly long training time (∼20 hours on a 16GB, 2.66GHz processor for a single model)—to first find the parameters (encoding, number of c_topics), then the c_topics themselves, and finally, to learn the model that has the best performance. (However, using the trained dLDA model to predict c_topic contributions for a new patient is very fast—under a second on a general purpose laptop computer.)
- Advantages:
  - An effective process to learn representation from gene expression data, as a meaningful probability distribution over the genes.
  - The learned representation from the gene expression data improves survival prediction, over standard methods, in:
    - Different cancer types: Breast and Kidney.
    - Different gene expression data types: Microarray and mRNASeq.
    - Different survival prediction algorithms: Cox, Regularized-Cox and MTLR.
  - Our combined approach for feature learning and survival prediction (dLDA + MTLR) archives strong concordance scores compared to standard survival models across different cancer types.

## 7 Conclusion

Table 2 shows that our proposed model, which uses MTLR to learn a model involving various types of derived GE features (dLDA c_topics and/or SuperPC+), has the best concordance, in two datasets representing different types of cancer, and two different gene expression platforms (micro-array and mRNAseq). That table shows that adding GE features improves survival prediction and that including both dLDA c_topics and SuperPC+ principle components gives the most improvements across held-out datasets. We also found that the “framework” that produced the best model in METABRIC, was also the best in the Pan-kidney KIPAN dataset, which shows the robustness of our proposed prediction framework. Moreover the c_topics extracted by our dLDA procedure (inspired by topic modeling) can be interpreted as collections of over-expressed or under-expressed gene sets; further analysis is needed to discover and validate the biological insights from these c_topics. Our results show that our novel survival prediction model—learning a MTLR survival model based on our derived GE features (dLDA c_topics and SuperPC+ components)—leads to survival prediction models that can be better than standard survival models. We anticipate that others will find this dLDA+MTLR approach (and code at https://github.com/nitsanluke/GE-LDA-Survival) helpful for their future tasks.

## A Details about the algorithms

**Fig 8:**
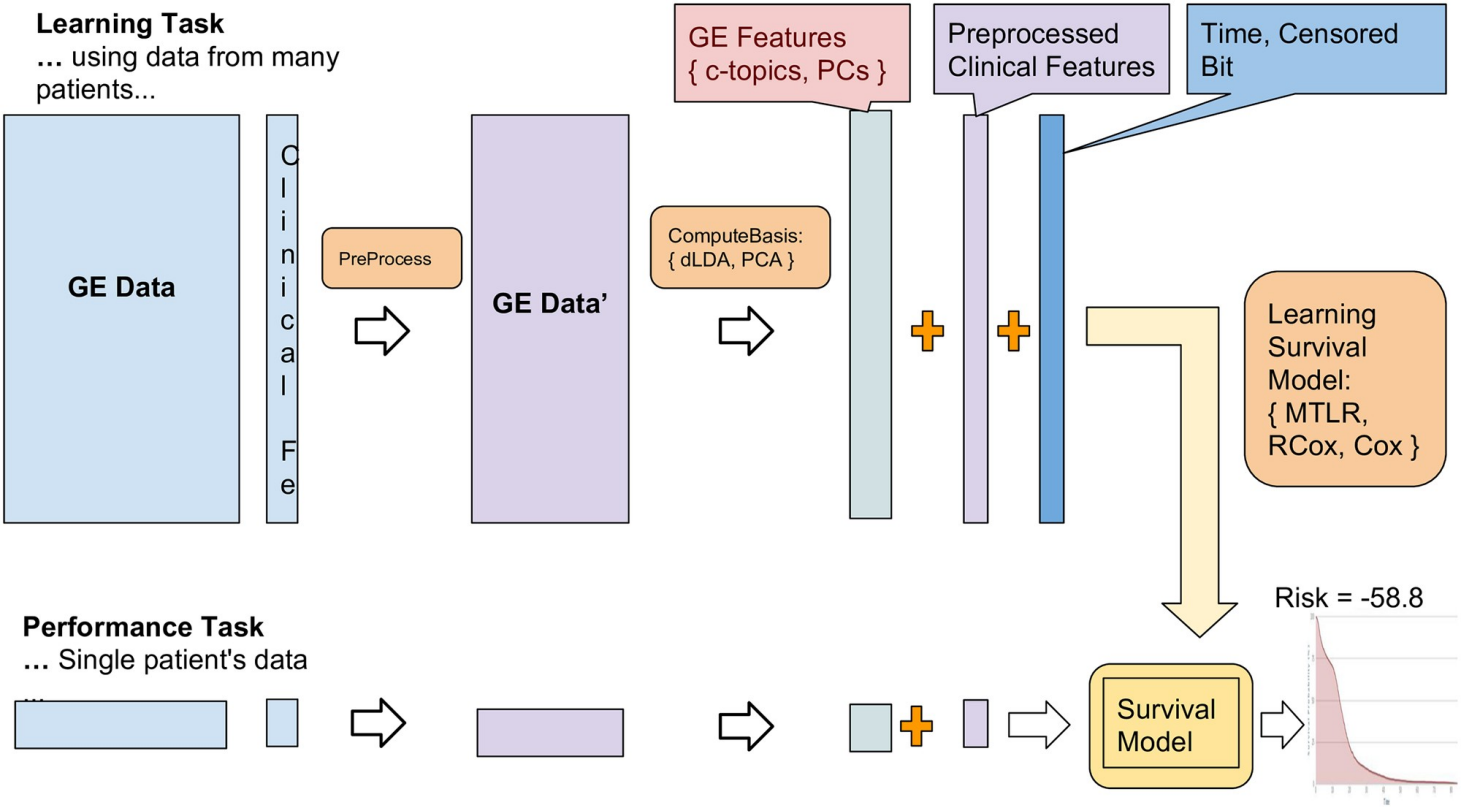
Simplified flow diagram describing the overall prediction process, including both the learning task and the performance task.

Section 4 gave a high-level overview of the important parts of the learning, and performance, systems; see also Fig 8. This appendix completes that description. In particular, it summarizes the components of the learning and performance systems—each shown as a rounded-rectangle in Figs 3 or 6—roughly in a top-to-bottom fashion. Appendix A.1 describes the PreProcess(⋯) routine that preprocesses the training data (both gene expression and clinical features), and the related PreProcess’(⋯) routine, used by USM, to preprocess a novel instance. Appendix A.2 then gives many details about ComputeBasis_[*ρ*=dLDA]_(⋯) that computes the set of “c_topic − genes” distributions, given gene expression values (and some additional information)—extending the high-level description in routine in Section 4.1.1. Appendix A.3 describes the UseBasis_[*ρ*=dLDA]_ routine that uses these c_topic-genes distributions to map each patient’s gene expression profile into that patient’s specific “c_topic—distribution”; see Fig 5. Appendix A.4 presents ComputeBasis_[*ρ*=PCA]_ and UseBasis_[*ρ*=PCA]_ techniques, to deal with the other approaches for reducing the dimensionality, PCA. Finally Appendix A.5 describes two related standard survival analysis methods: Cox [26], and Ridge-Cox (RCox) [33]. (Section 2.1.1 presented another approach, based on the more recent MTLR approach to survival analysis.)

### A.1 PreProcess and PreProcess’

The PreProcess process (used by LSM in Fig 3) applies various standard “normalizations” and simple “corrections” to the training data—both raw clinical features, and gene expression values. For the clinical features *X*_*CF*_, PreProcess produces a normalized dataset without any missing values, ready for the subsequent steps in the pipeline—see the orange-lines in Fig 3. This uses the standard steps: (1) impute missing real (resp., categorical) values for a feature with the mean (resp., mode) of the observed values for that feature; and (2) binarizing each categorical variable (aka “one-hot encoding”)—*e.g*., we encoded the 12-valued “Histological type” using twelve bits: *e.g*., *Invasive Tumor* is [1, 0, 0, 0, 0, 0, 0, 0, 0, 0, 0, 0]. For the gene expression data *X*_*GE*_, PreProcess applies the following steps: (1) As we want to deal with the log of the initial gene expression value, we first log_2_-transformed the data, if necessary. (Below we use “gene expression” to refer to this transformed value.) (2) Then translate all expression values into their “common z-scores”. It first computes the (common) mean and standard deviation over all the genes from the entire *X*_*GE*_ dataset: Let $e_{i}^{j}$ be the expression value of probe/gene *g*_*i*_ of patient *j*, then compute the common mean $\hat{\mu }=\frac{1}{n}\sum_{i,j}e_{i}^{j}$ (where *n* = 1,981×49,576 is the total number of entries for METABRIC), and the variance ${\hat{\sigma }}^{2}=\frac{1}{\left(n-1\right)}\sum_{i,j}{\left(e_{i}^{j}-\hat{\mu }\right)}^{2}$. We then use the Z-score transformation of each entry: $z_{i}^{j}=\frac{\left(e_{i}^{j}-\hat{\mu }\right)}{\hat{\sigma }}$. Notes: (a) this standardization is done prior to dividing the data into train and validation sets. (b) Using z-scores based on only a single gene would not be able to identify which genes did not vary much, as (after this transform) all genes would vary the same amount. (3) PreProcess then removes the genes that do not vary much, removing a gene *i* iff its $z_{i}^{j}$ values are all within the first standard deviation—*i.e*., if $\forall j\;z_{i}^{j}\in \left[-1,+1\right]$.

This filtering process is motivated by the assumption that any gene whose expressions does not change much across multiple patients, is unlikely to be directly related to the disease, while the genes that contribute, typically have significant variations in their expression levels across patients. While this filtering procedure is unsupervised, we anticipated that it would retain the genes that have the most prognostic ability. This was confirmed as we found that this process does not eliminate any of the “top” 100 probes in the METABRIC data (these are the probes with the 100 highest concordance values); see [13, Table 1]. In METABRIC, this filtering procedure eliminates 27,131 of the original 49,576 probes, leaving only 22,445 probes—*i.e*., a ≈54.7% reduction in the number of features.

Later, the performance system USM will need to apply these pre-processing steps to a novel instance—in particular, for each clinical feature, it will need to know the mean (or median) value, for imputation. Similarly, it will need to transform each gene expression values into an integer; this requires knowing the global mean $\hat{\mu }$ and ${\hat{\sigma }}^{2}$ values to produce the *z*-values $\left\{z_{i}^{j}\right\}$ values, We include all of these values in the Ω term, which is output by the PreProcess subroutine. This Ω is one of the inputs to the PreProcess’ process, within USM, which applies these pre-processing steps to a novel instance encoded by its *x*_*GE*_ and *x*_*CF*_ features. Note finally that neither PreProcess nor PreProcess’ use the labels (survival times).

### A.2 ComputeBasis_[*ρ*=dLDA]_

As shown by the blue lines in Fig 3, the ComputeBasis process takes as input a pre-processed version of the labeled dataset that was input to LSM: the PreProcessed gene expression data $X_{GE}^{\prime }$ and clinical features $X_{CF}^{\prime }$, with their associated labels (*Lbl*). This process produces the “basis” set, of type *ρ*. This subappendix will focus on *ρ* = dLDA.

**Algorithm 1** ComputeBasis_[*ρ*=dLDA]_ algorithm

1: **function** ComputeBasis_[*ρ*=dLDA]_($X_{GE}^{\prime },\;X_{CF}^{\prime },\;Lbl$)     ⊳ Returns a set of c_topics

2:  $X_{GE}^{\prime \prime }$ ≔ Discretize($X_{GE}^{\prime }$)

3:  **for** t in {Enc_A, Enc_B} **do**

4:   *GE*_*t*_ ≔ Encode-GE(t, $X_{GE}^{\prime \prime }$)

5:   [*GE*_*t*,1_, *GE*_*t*,2_, …, *GE*_*t*,5_] ≔ Partition(*GE*_*t*_)

6:   % Notation: *GE*_*t*,−*i*_ = *GE*_*t*_ − *GE*_*t*,*i*_

7:   % $X_{CE,i}^{\prime }$ = clinical features;

8:   **for** K in (5, 10, 15, …, 150) **do**

9:    **for** i = 1:5 **do**

10:     % Find LDA “basis set” (set of c_topics)

11:     ${\bar{\beta }}_{t,K,i}$ ≔ Compute_dLDA(*GE*_*t*,−*i*_, K)

12:

13:     % Project the hold-out set onto this basis set,

14:     % encoding each patient as a K-tuple of values

15:     *GE*_*Topic*_*t*,*K*,*i*_ ≔ Use_dLDA(*GE*_*t*,*i*_, ${\bar{\beta }}_{t,K,i}$)

16:

17:     % Learn a Cox model for this encoding, value of *K*, and fold *i*

18:     % using both c_topics and the clinical features

19:     % LearnCox & PredictCox are based on [26]

20:     *w*_*t*,*K*,*i*_ ≔ LearnCox($\left[\;GE\_Topic_{t,K,i},\;X_{CF,i}^{\prime }\;\right],\;Lbl_{i}$)

21:

22:     % Evaluate model on the hold out set

23:     % Using evaluation measures concordance and likelihood

24:     *c*_*t*,*K*,*i*_ ≔ Concordance(PredictCox($w_{t,K,i},\;\left[GE_{t,i},\;X_{CF,i}^{\prime }\right]$), *Lbl*_*i*_)

25:     *l*_*t*,*K*,*i*_ ≔ Average{Likelihood(${\bar{\beta }}_{t,K,i}$, *GE*_*t*,*i*_)}

26:

27:    ${\bar{c}}_{t,K}$ ≔ Average{*c*_*t*,*K*,*i*_}

28:    ${\bar{l}}_{t,K}$ ≔ Average{*l*_*t*,*K*,*i*_}

29:

30:   % Find t* (encoding scheme), with the highest concordance

31:   t* ≔ $arg\;{\text{max}}_{t}\left\{\;{\bar{c}}_{t,K}\;\right\}$

32:   % Selecting K*

33:   $\hat{K}\;=\;arg\;{\text{max}}_{K}\left({\bar{l}}_{t^{*},K}\right)$

34:   $K^{*}=\text{arg}\;{\text{max}}_{K\;s.t.\;{\bar{l}}_{t^{*},\hat{K}}\;-\;\hat{\sigma }\left(l_{t^{*},\hat{K}}\right)\;\leq \;\;{\bar{l}}_{t^{*},K}}\left\{{\bar{c}}_{t^{*},K}\right\}$ % Break ties giving priority to small K’s

35:

36:   **return** Compute_dLDA(*GE*_t*_, K*)

As shown at the bottom of Algorithm 4, ComputeBasis returns the results of Compute_dLDA(*GE*_t*_, K*), which are a set of K* c_topic–distributions, based on its input *GE*_t*_, which encodes the gene expression values ($X_{GE}^{\prime }$) as non-negative integers. This means ComputeBasis must first (1) transform its input real-valued gene expression values $X_{GE}^{\prime }$ into non-negative integers *GE*_t*_, and (2) determine the appropriate number of c_topics $K^{*}\in Z^{+}$. Task (1) has two parts: (1a) Line 2 first discretizes the real-valued *X*_*GE*_ into (positive and negative) integers Z. (1b) The next part of the subroutine determines the best way to transform those integers into *non-negative integers* $Z^{\geq 0}$. Below we describe these three steps, followed by (3) a description of Compute_dLDA.

#### (1a) Discretize subroutine

Recall first that the PreProcess routine already translated the real-valued *X*_*GE*_ gene expression values into z-scores $X_{GE}^{\prime }$, and excluded every genes whose values here all were in (−1, +1). To simplify the notation, view $X_{GE}^{\prime }=\left\{z_{i}^{j}\right\}$. The Discretize routine first assigns each $z_{i}^{j}\in \;\left(-1,1\right)$ to 0. For the remaining “non-trivial” standardized gene expression values $z_{i}^{j}$’s (outside the first standard deviation) of each gene: Letting $Z_{i}^{+}\;=\;\left\{\;z_{i}^{j}\;|\;z_{i}^{j}\geq 1\;\right\}$ be the non-trivial positive values, we divide $\Delta_{i}^{+}\;=\;\text{max}\left\{Z_{i}^{+}\right\}\;-\;\text{min}\left\{Z_{i}^{+}\right\}$ into 10 regions, of size $\Delta_{i}^{+}/10$ each and identify each positive $z_{i}^{j}$ with the index ∈{ 1, 2, …, 10} of the appropriate bin. We similarly divide the non-trivial negative expression values $Z_{i}^{-}\;=\;\left\{z_{i}^{j}\;|\;z_{i}^{j}\leq -1\;\right\}$ into their 10 bins, based on $\Delta_{i}^{-}\;=\;\text{max}\left\{Z_{i}^{-}\right\}-\min\left\{Z_{i}^{-}\right\}$ and each negative $z_{i}^{j}$ is identified with the index ∈{ -1, -2, …, -10} of the appropriate bin; see Fig 4. (Of course, the actual divisions are specific to the different genes; this figure just shows a generic split.) In general, we let $b_{i}^{j}$ be the integer bin index associated with gene *g*_*i*_ for subject *j*.

Notes: (1) We initially tried to discretize the values into the bins associated with the standard deviation, in general. However, we found this did not work well. (2) ComputeBasis also returns these ${\left\{\Delta_{i}^{+},\Delta_{i}^{-}\right\}}_{i}$ values, as part of the encoding –*i.e*., along with ${\bar{\beta }}_{GE}$—and UseBasis will later use this information to discretize its real-valued gene expression input. We did not show this detail, to avoid overcluttering the text and images.

#### (1b) Transform to non-negative integers

While Discretize mapped each gene expression value $z_{i}^{j}$ to an integer $b_{i}^{j}$, the Compute_dLDA routine requires *non-negative* values. Section 4.1.1 discussed two ways to deal with this: using either encoding Enc_A versus Enc_B; see bottom of Fig 4. ComputeBasis uses internal cross-validation to determine which of these is best, t*, along with the number K* of c_topics; see below. (In general, we will let *GE*_*t*_ refer to the *t*-encoding of the gene expression values.)

#### (2) Finding optimal K*, t*

As noted, the Compute_dLDA algorithm also needs to know the number of c_topics K* to produce. Rather than guess an arbitrary value, ComputeBasis instead uses (internal) cross-validation to find the best value for *K*, over the range *K* ∈ {5, 10, 15, …, 150}. For each technique *t* ∈ {Enc_A, Enc_B} and each of the 30 values of *K*, ComputeBasis first computes the dLDA model over the training set, using Compute_dLDA (for that encoding and number of c_topics); it then used these and the (preprocessed) clinical features ($X_{CF}^{\prime }$) as covariates, along with the survival labels *Lbl*, to learn a Cox model [26]—see Algorithm 1, lines 9–20. Note it does this in-fold—using 4/5 of the training set to learn the dLDA c_topics and the Cox model, which is evaluated by computing the concordance (based on this learned model) on the remaining 1/5 (line 24).

As noted above, we need to determine (1b) which is the best discretization t*, Enc_A or Enc_B, and (2) what is the appropriate K* for that technique. To answer the first question, ComputeBasis picked the encoding technique t* that gave the highest cross-validation concordance from all the (30 × 2) combinations (see Algorithm 1, line 31). Secondly, after deciding on a encoding scheme, it sets $\hat{K}$ to be the value with the largest (cross-validation) likelihood, then selects the set of *K*’s that are smaller than $\hat{K}$ and whose cross-validation likelihood scores are within the first standard deviation of the $\hat{K}$’s; see Algorithm 1, line 33. From these candidates, it selected the K* that gives essentially the highest concordance (see Algorithm 1, line 34). Empirically, we found that the internal cross-validation concordance scores was fairly flat over the critical region—*e.g*., *K* ∈ {20, ‥, 35} for METABRIC—before dipping to smaller values for larger value of K, presumably due to overfitting. This is why we are confident that the upper limit, of 150 topics, is sufficient. Once it finds the best K* and the encoding technique t*, ComputeBasis then runs Compute_dLDA on the t*-encoded (preprocessed) training gene expression data *GE*_t*_, seeking K* c_topics; this is ${\bar{\beta }}_{GE}$ “basis”. This routine also returns the $\left\{\Delta_{i}^{\pm }\right\}$ values used to produce the discretized values, *GE*_*t*_.

#### (3) Compute_dLDA

The Compute_dLDA(*GE*_*t*_, *K*) process, based on Blei *et al*. [17], computes *K* c_topics, based on the preprocessed, discretized gene expression data *GE*_*t*_, as well as the number of latent c_topics *K*; it then returns *K* c_topics–distributions, each ≈50,000-parameters of the Dirichlet distribution (for METABRIC), corresponding to a line of the ${\bar{\beta }}_{GE}$ shown in Fig 5. (Each point here corresponds to its estimate of the posterior *β*_*GE*_, conditioned on the observed gene expression values.) This routine also uses the Dirichlet prior for the patient–c_topics distribution; here we used the symmetric Dirichlet(*α*, …, *α*) for some *α* ∈ ℜ^>0^. (As there are *K* c_topics; we view this as a vector *α***1**_*K*_.) We experimented with several values *α* ∈ {0.01, 0.1, 0.5, 1.0}, but found that the prior did not make much difference, since we allowed the model to estimate the prior internally. We therefore set *α* = 0.1.

This routines also needs to set the priors for the *K* different c_topic–gene_expression distributions $\beta_{GE}^{\left(*\right)}\left[i,:\right]=\left[\beta_{GE}^{\left(*\right)}\left[i,1\right],\ldots ,\beta_{GE}^{\left(*\right)}\left[i,N\right]\right]$, for *i* = 1‥*K*, each sweeping over the *N* genes. Here, we use the prior $\beta_{GE}^{\left(*\right)}\left[i,j\right]\;=\frac{1}{N}+\delta$ where *δ* ∼ *U*[0, 1/*N*^2^]—*i.e*., *δ* is sampled from the uniform distribution over the interval [0, 1/*N*^2^]. This LDA learning process [17] uses the data in *GE*_*t*_ to compute the posterior distribution {*β*_*GE*_[*i*,:]}_*i*_ for each of these *K* c_topics—revealing *GE*_*t*_’s intrinsic structure. Recall these are just the parameters for Dirichlet distribution; note they must be positive, but do not add up to 1. The Compute_dLDA returns the expected values of the gene expression values drawn from this posterior distribution: ${\bar{\beta }}_{GE}\left[i,j\right]=\frac{\beta_{GE}\left[i,j\right]}{\sum_{j^{\prime }}\beta_{GE}\left[i,j^{\prime }\right]}$. Here, the probability values for each c_topic ${\bar{\beta }}_{GE}\left[i,:\right]$ add up to 1. We will let ${\bar{\beta }}_{GE}=\left\{{\bar{\beta }}_{GE}\left[i,:\right]\right\}$ refer to the entire “matrix”.

### A.3 UseBasis_[*ρ*=dLDA]_

Once LSM has learned the c_topics (${\bar{\beta }}_{GE}$) for the best K* and best encoding technique t*, we can then compute the c_topic distribution for a new patient (based on her gene expression *x*_*GE*_); see Figs 3 and 6. This will call UseBasis, which in turn runs Use_dLDA (the *LDA inference procedure*) on the preprocessed gene expression data $x_{GE}^{\prime }$ of the current patient to compute the individual topic contributions for this patient [17]. The inference procedure determines the posterior distribution of the **patient-c_topic** Dirichlet distribution $\Theta \left(x_{GE}^{\prime }\right)\;=\;\left[\;\theta_{1}\left(x_{GE}^{\prime }\right),\;\theta_{2}\left(x_{GE}^{\prime }\right),\;\ldots ,\;\theta_{K^{*}}\left(x_{GE}^{\prime }\right)\;\right]\;\in \;{ℜ}_{+}^{K^{*}}$, where each $\theta_{j}\left(x_{GE}^{\prime }\right)\;\in \;{ℜ}_{+}$ quantifies how much of this patient’s gene expression is from the *j*^*th*^ c_topic (using the posterior mean probabilities of the c_topics, ${\bar{\beta }}_{GE}$).

This process reduces the ≈20 000-dimension gene expression values to a very small K*-dimensional c_topics representation—*e.g*., L* = 30. These low-dimensional feature vectors are then used in the survival prediction algorithms to predict survival times/risk.

### A.4 ComputeBasis_[*ρ*=PCA]_ and UseBasis_[*ρ*=PCA]_

The previous subappendix described one way to reduce the dimensionality of the data—to transform each patient’s 20,000-tuple to a more manageable *K*-tuple—there based on topic modeling ideas. There have been many other feature selection methods proposed for survival prediction using gene expression data, such as hierarchical clustering, univariate gene selection, supervised PCA, penalized Cox regression and tree-based ensemble methods [34]. Some of these techniques first apply a procedure to reduce the dimensionality of the data, based on feature selection, feature extraction or a combination of both, while others, such as random survival forests [9] and L1-penalized Cox [35], include internal feature selection. As we wanted to compare our dLDA approach to other dimensionality reduction techniques, we chose an extension to the principle component analysis called supervised principal component analysis (SuperPC) [36], instead of other regularization techniques.

This algorithm first calculates the univariate Cox score statistic of each individual gene against the survival time, then retains just the subset of genes whose score exceeds a threshold, determined by internal cross-validation. Then it computes PCA on the dataset containing only those selected genes, then projects each patient onto the first one (or two) components. The main disadvantage of the SuperPC algorithm is that the individual genes selected from the univariate selection process might not perform the best in a multivariate (final) model, perhaps because many of these top-ranked genes may be highly correlated with one another –*i.e*., it would be better having a more “diverse” set of genes [34, 37]. Instead, we use a variant, called SuperPC+, that initially applies PCA on the *normalized* gene expression data after the constant genes are removed; see PreProcessin Appendix A.1). The PCA transformation projects the initial “raw” features into a different space, which then can be used to select the top components based on the univariate Cox regression. Note this SuperPC+ is (still) computationally efficient, as it is based on PCA, which is efficient: Even though gene expression data is high dimensional (*p* ≫ *n*, where *p* is the number of genes and *n* is the number of instances), the rank of the GE matrix will be (at most) min{*p*, *n*} = *n*. Therefore, PCA can be performed without many computational restraints on the whole gene expression dataset, as here the PCA time complexity is *O*(*n*^3^). After performing PCA on the GE dataset, we can then identify the most important principal components by computing a Cox score statistic for the univariate association between each principal component and the survival time. In our experiments, we select the threshold *η* for the p-value of the Cox score by internal cross-validation (wrt concordance), and retained all PCs having a p-value lower than this *η*—finding *η* = 5e-4 for the METABRIC dataset and *η* = 5e-2 for KIPAN. These selected PC components form the basis set *B*_*GE*_[*ρ* = PCA].

UseBasis_[*ρ*=PCA]_ is simply the projection of the gene expression data into the chosen PC components. This gives us a low dimensional feature representation of the original gene expression data to feed into the survival prediction algorithms.

### A.5 Cox models: LearnModel_[Ψ=Cox]_, LearnModel_[Ψ=RCox]_

The Cox regression model’s [26] hazard function over time *t*, for an individual described by *x*, is the product of two components:

$$\begin{array}{c} h_{W}\left(\;t\;|\;x\;\right)\;=\;h_{0}\left(t\right)\times \text{exp}\left(x^{T}W\right) \end{array}$$ (3)

where the baseline hazard *h*_0_(*t*) is independent of the covariates *x* and the covariates are (independently) multiplicatively related to the hazard, based on a (learned) *W*. This formulation simplifies modeling of the hazard function by limiting the contribution of the “time” variable *t* to the baseline hazard *h*_0_(⋅), which means the hazard ratio (HR) between two patients

$$\begin{array}{c} \text{HR}\left(x_{1},\;x_{2}\right)\;=\;\frac{h(\;t\;|\;x_{1}\;)}{h(\;t\;|\;x_{2}\;)}\;=\;\frac{h_{0}\left(t\right)\;\text{exp}\left(x_{1}^{T}W\right)}{h_{0}\left(t\right)\;\text{exp}\left(x_{2}^{T}W\right)}\;=\;\text{exp}\left({\left(x_{1}-x_{2}\right)}^{T}W\right) \end{array}$$

does not depend on time and is linear (proportional) in the exponent. To estimate the coefficients of the model, Cox [26] proposed a partial likelihood technique that eliminates the need to estimate the baseline hazard. This procedure allows the Cox proportional hazards model to be semi-parametric, by only using the survival times to rank the patients [32]. We can compute partial likelihood with all patients—both censored and uncensored:

$$L_{c}\left(W\right)=\prod_{i=1}^{N}(\frac{\text{exp}({x_{i}}^{T}W)}{\sum_{k\in R(y_{i})}\text{exp}\left({x_{k}}^{T}W\right)}{)}^{\delta_{i}}$$ (4)

- $R(y_{j})$ is the risk set at time *y*_*j*_, which are the indices of individuals who are alive and not censored before time *y*_*j*_
- [*x*_*i*_, *y*_*i*_, *δ*_*i*_] describes the *i*^*th*^ subject, where
   *x*_*i*_ = vector of covariates
   *y*_*i*_ = (survival or censor) time
   *δ*_*i*_ = censor bit (0 for censored; otherwise 1)
- *N*—total number of patients in the cohort
- *W*—coefficients (to be learned)

Note that only the uncensored likelihoods contribute directly, since for censored instances *δ*_*i*_ = 0. Therefore the censored observations are only utilized in the denominator when summed over the instances in a risk set. In essence, the partial likelihood only uses the patient’s death times to rank them in the ascending order to find the risk sets and does not use the exact times explicitly [32]. Hence, the coefficients estimated by maximizing the partial likelihood depend only on the ordering of the patient’s death times and the covariates, allowing for an implicit optimization for good concordance of the risk score. An in-depth study on the Cox proportional hazard model has revealed that the partial likelihood proposed by [26] is approximately equivalent to optimizing concordance [32].

There are several extensions of the basic Cox proportional hazards model: some extend the initial model estimating the baseline hazard and others are based on the regularization methods imposed on the coefficients (*W*). Generally, regularization based on LASSO, ridge penalty or the elastic-net regularization (which allows both L1 and L2 penalties) are adopted to reduce overfitting. In our work, we use the glmnet R package [33] with ridge penalty (by setting *α* = 0 in the glmnet function); here called RCox. We selected ridge penalty based on the internal cross validation. We found that concordance results using models with ridge penalty were better than those having no regularization (LASSO, elastic-net).

## B Foundations

### B.1 Evaluation: Concordance Index (CI)

This “CI” evaluation applies to any model that assigns a real number—a “risk score”—to each instance *f*(⋅). It considers all pairs of “comparable” instances, and determines which is predicted (by the risk model *f*(⋅)) to die first, and also who actually died first. CI is the proportion (probability) of these pairs of instances whose actual pair-wise survival ordering, matches the predicted ordering, with respect to *f*(⋅):

$$\begin{array}{c} \text{CI}\left(f\right)=\frac{1}{\left|\Psi \right|}\sum_{(i,j)\in \Psi }I\left[\;f\left(x_{i}\right)>f\left(x_{j}\right)\;\right] \end{array}$$ (5)

where *I*[*ϕ*] is the indicator function, which is 1 if the proposition *ϕ* is true, and 0 otherwise. A pair of patients is “comparable” if we can determine which died first –*i.e*., if both are uncensored, or when one patient is censored after the observed death time of the other; this corresponds to the set of ordered pairs of indices Ψ. This CI(*f*) score is a real value between 0 to 1, where 1 means all comparable pairs are predicted correctly. CI can be viewed as a general form of the Mann–Whitne–Wilcoxon statistic and is similar to the Area Under the ROC (Receiver Operator Curve), AUC, of classification problems [32].

### B.2 Evaluation: D-calibration

The concordance index is a discriminatory measure, which is relevant, for example, when deciding which patient with liver failure will die first without a transplant. By contrast, *calibration* measures the deviation between the observed and the predicted event time distributions. While this is not meaningful if we only have a risk score (*e.g*., as produced by the basic Cox Proportional Hazard function), this deviation can be computed for a *survival distribution*, like ones produced by the MTLR survival prediction tool, or the Cox+KF system—which extends the standard Cox model by using the Kalbfleisch-Prentice estimator to produce the baseline hazard function *h*_0_(*x*) in Eq 3; see [11]. In general, this calibration involves computing the difference between the predicted versus observed probabilities in various subgroups—*e.g*., if the predicted probability of surviving at least *t* = 2576 days is 0.75 for some subgroup, then we expect to observe around 75% of these patients to be alive at this time *t*.

**Fig 9:**
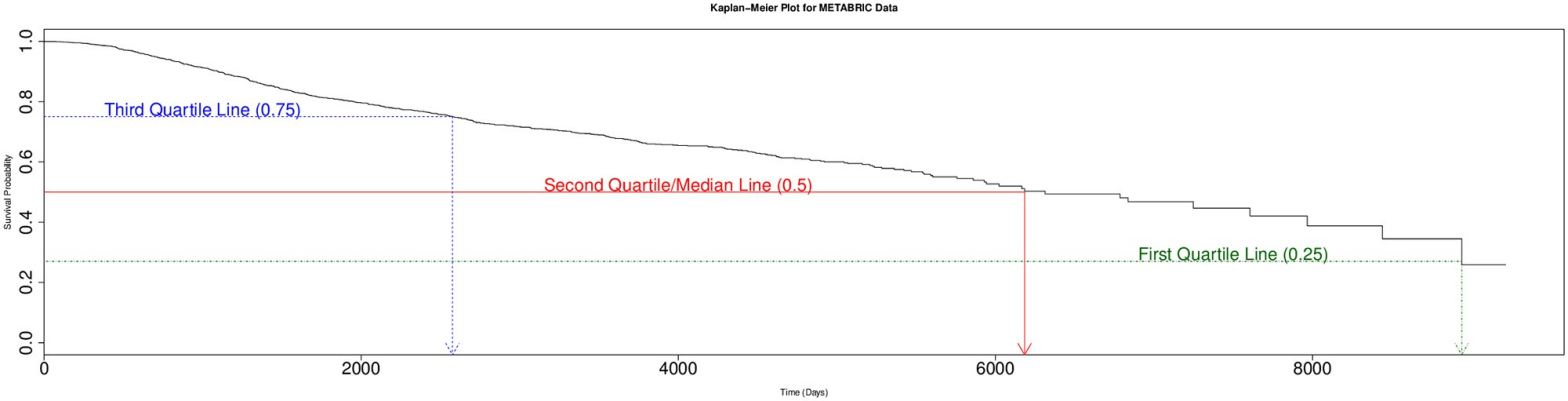
Kaplan–Meier survival function from METABRIC (training) data. (We only use the quartiles for pedagogic purposes.).

We consider a novel measure of the calibration of such survival curves, called D-calibration (“D” for “Distribution”) [16]. To motivate this, consider a standard Kaplan-Meier (KM) [8] plot shown in Fig 9, which plots the set of points (*t*, KM(*t*))—*i.e*., it predicts that the KM(*t*) fraction of patients will be alive at each time *t* ≥ 0. Hence, the point (6184 days, 0.50) means the median survival time of the cohort is 6184 days; see Fig 9(solid line). We will use KM^−1^(*p*) to be the time associated with the probability *p*—technically, KM^−1^(*p*) is the earliest time when the KM curve hits *p*; hence KM^−1^(*p*) (0.5) = 6184 days. If this plot is D-calibrated, then around 50% of the patients (from a hold-out set, not used to produce the KM curve) will be alive at this median time. So if we (for now) ignore censored patients, and let *d*_*i*_ be the time when the *i*^*th*^ patient died, consider the *n* values of {KM(*d*_*i*_)}_*i*=1‥*n*_. Here, we expect KM(*d*_*i*_) > 0.5 for 1/2 of the patients. Similarly, as the curve includes (2576 days, 0.75) and (8941 days, 0.25), then we expect 75% to be alive at 2576 days, and 25% at 8941 days; see Fig 9. Collectively, this means we expect 25% of the patients to die between KM^−1^ (1.0) = 0 days and KM^−1^ (0.75) = 2576 days, and another 25% between KM^−1^ (0.75) and KM^−1^ (0.5), etc. These are the predictions; we can also check, to see how many people actually died in each interval: in the first quartile (between 0 and 2576 days), in the second (between 2576 and 6184 days), in the third (between 6184 and 8941 days), and the fourth (after 8941 days). If the KM plot is “correct”—*i.e*., is D-calibrated—then we expect 1/4 of the patients will die in each of these 4 intervals. The argument above means we expect 1/4 of the {KM(d_*i*_)} values to be in the interval [0, 0.25], and another quarter to be in [0.25, 0.5], etc. Stated more precisely,

$$\begin{array}{c} \text{the}\;\text{values}\;\text{of}\;\left\{\;\text{KM}\left(\;d_{i}\;\right)\;\right\}\;\text{are}\;\text{uniformly}\;\text{distributed.} \end{array}$$ (6)

A single KM curve is designed to represent a cohort of many patients. The MTLR system, however, computes a different survival curve for each patient—call it *Pr*_*i*_(·) = *Pr*_*W*_ (· | **x**_*i*_) (from Eq 1). But the same ideas still apply: Each of these patients has a median predicted survival time—the time $Pr_{i}^{-1}\left(0.5\right)$ where its *Pr*_*i*_(⋅) curve crosses 0.50.

By the same argument suggested above, we expect (for a good model *W*) that 1/2 of patients will die before their respective median survival time—$d_{i}\leq Pr_{i}^{-1}\left(0.5\right)$; that is, |{*i*: *Pr*_*i*_(*d*_*i*_) ≤ 0.5}| ≈ *n*/2. Continuing the arguments from above, we therefore expect the obvious analogue to Eq 6:

$$\begin{array}{c} \text{the}\;\text{values}\;\text{of}\;\left\{Pr_{i}\left(\;d_{i}\;\right)\right\}\;\text{are}\;\text{uniform.} \end{array}$$ (7)

We can now test whether a model is D-calibrated by using the Hosmer-Lemeshow (HL) [30] goodness-of-fit test, which compares the difference between the predicted and observed events in the event subgroups:

$$\begin{array}{c} \text{HL}(\;[\begin{array}{c} \left[N_{1},\;P_{1},\;E_{1}\right]\\ \cdots \\ {}[N_{G},\;P_{G},\;E_{G}] \end{array}])\;=\;\sum_{g=1}^{G}\frac{{\left(E_{g}-P_{g}\right)}^{2}}{N_{g}\pi_{g}\left(1-\pi_{g}\right)} \end{array}$$ (8)

where *G* is the number of subgroups (here 4), where the *g*^*th*^ subgroup has $N_{g}\in Z^{+}$ events, with the empirical number of events $E_{g}\in Z^{+}$ (which here is *N*/*g*, for *N* = ∑_*g*_ *N*_*g*_ total patients), the corresponding predicted number of events in each group $P_{g}\in Z^{\geq 0}$, and $\pi_{g}=\frac{N_{g}}{N}$ (which here is $\frac{1}{G}$) is the proportion of the *g*^*th*^ subgroup. Under the null hypothesis (Eq 7), this HL statistics follows a Chi-Square distribution with *G* − 2 degrees of freedom. If the predicted and empirical event rates are similar for the subgroups, the test statistic will fail to reject the null hypothesis, providing evidence that the model’s predictions are well D-calibrated—*i.e*., large p-values from the test statistic suggest not rejecting the null hypothesis).

Notes: (1) This evaluation criterion only applies to models that produce survival distributions, which means it directly applies to the MTLR models. For the Cox and RCox models, we used the Kalbfleisch-Prentice baseline hazard estimator [11] to produce personalized survival curves. (2) To provide more precise evaluation, rather than using 4 bins (quantiles), we mapped the *Pr*_*i*_(*d*_*i*_) probabilities into 20 bins: [0, 0.05); [0.05, 0.1), …, [0.95, 1.0]. (3) This analysis deals only with uncensored data; Haider *et al*. [16] discusses how to cope with censored data.

## C Additional results

This appendix presents additional results: First, Appendix C.1 evaluates the Latent process decomposition (LPD) method, then Appendix C.2 provides D-calibration results of our various models.

### C.1 Latent process decomposition (LPD) for microarray feature extraction

Rogers *et al*. [19] introduced LPD as a topic model adaptation for microarray data. We experimented with LPD (on METABRIC data) to derive genetic features and used them along with the clinical features for comparison. We used internal cross-validation for LPD to find the optimal number of latent processes for the METABRIC data—and found that 10 was best.

We then used the model based on these 10 latent process; the resulting concordance results, on the hold-out dataset, was 0.6915 (Cox), 0.6077 (RCox) and 0.6995 (MTLR). Comparing this to the “B” and “C” rows of Table 2, we see that our dLDA approach performs better than this complex adaptation of the LDA model for microarray data, for the survival prediction task—*i.e*., dLDA produces better features from the gene expression data.

There are two other reasons to prefer our dLDA-approach: (1) LPD has large time and memory requirements. (2) Moreover as our dLDA directly uses the LDA model, it can utilize all available off-the-shelf implementations, across several technology platforms with efficient and scalable implementation [38].

### C.2 D-calibration results

Table 3 shows the D-calibration results for all of the domain-independent experiments we ran—*i.e*., excluding the “E” and “F” rows from Table 2, which used features that were specific to breast cancer. We see that the results were D-calibrated (*i.e*., had a HL p-value > 0.05) in all 12 situations, for METABRIC and KIPAN—for all feature groups {A, B, C, D}, and all 3 learning algorithms {Cox, RCox, MTLR}. We note that we found that Cox failed this test on other datasets, including BRCA.
